# Phosphinous Acid–Phosphinito Tetra-Icosahedral Au_52_ Nanoclusters for Electrocatalytic Oxygen Reduction

**DOI:** 10.1021/jacsau.2c00517

**Published:** 2022-11-03

**Authors:** Shengli Zhuang, Dong Chen, Wai-Pan Ng, Dongyi Liu, Li-Juan Liu, Meng-Ying Sun, Tehseen Nawaz, Xia Wu, Yao Zhang, Zekun Li, Yong-Liang Huang, Jun Yang, Jun Yang, Jian He

**Affiliations:** † Department of Chemistry, The University of Hong Kong, Pokfulam Road, Hong Kong, P. R. China; ‡ State Key Laboratory of Synthetic Chemistry, The University of Hong Kong, Pokfulam Road, Hong Kong 999077, P. R. China; § State Key Laboratory of Multiphase Complex Systems, Institute of Process Engineering, Chinese Academy of Sciences, Beijing 100190, P. R. China; ∥ Department of Medicinal Chemistry, Shantou University Medical College, Shantou, Guangdong 515041, P. R. China

**Keywords:** cluster assembly, gold nanoclusters, hydrogen bonds, oxygen reduction reaction, phosphorus ligands

## Abstract

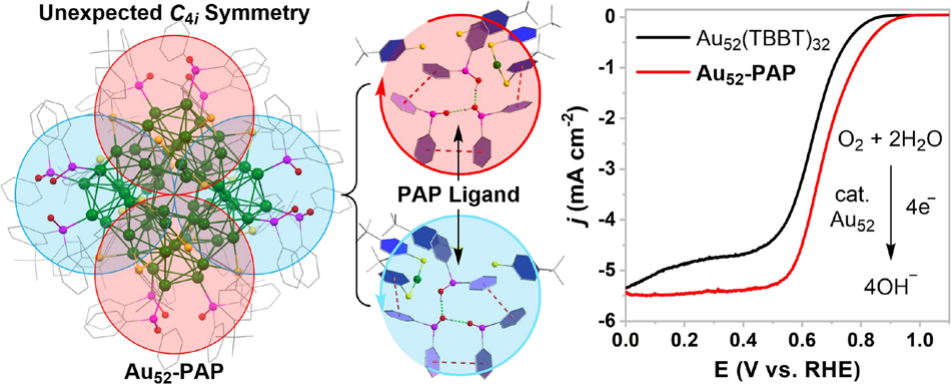

While the formation of superatomic nanoclusters by the three-dimensional assembly of icosahedral units was predicted in 1987, the synthesis and structural determination of such clusters have proven to be incredibly challenging. Herein, we employ a mixed-ligand strategy to prepare phosphinous acid–phosphinito gold nanocluster Au_52_(HOPPh_2_)_8_(OPPh_2_)_4_(TBBT)_16_ with a tetra-icosahedral kernel. Unlike expected, each icosahedral Au_13_ unit shares one vertex gold atom with two adjacent units, resulting in a “puckered” ring shape with a nuclearity of 48 in the kernel. The phosphinous acid–phosphinito ligand set, which consists of two phosphinous acids and one phosphinito motif, has strong intramolecular hydrogen bonds; the π–π stacking interactions between the phosphorus- and sulfur-based ligands provide additional stabilization to the kernel. Highly stable Au_52_(HOPPh_2_)_8_(OPPh_2_)_4_(TBBT)_16_ serves as an effective electrocatalyst in the oxygen reduction reaction. Density functional theory calculations suggest that the phosphinous acid–phosphinito ligands provide the most active sites in the electrochemical catalysis, with O* formation being the rate-determining step.

## Introduction

Ligand-protected gold nanoclusters have been extensively exploited in recent decades,^1−5^ since their unique geometric structures and molecule-like features are vital for the advancement of many impactful research fields, including bio-labeling,^6,7^ chemical sensing,^8,9^ and catalysis.^10−13^ Among many synthetic strategies currently used to prepare gold nanoclusters, protecting ligands play critical roles in the nanocluster formation, especially when it comes to constructing their kernel structures.^14^ While thiolate ligands are widely employed in the synthesis of gold nanoclusters containing single Au_13_ icosahedral,^15,16^ face-centered cubic (fcc),^17−21^ body-centered cubic (bcc),^22,23^ or hexagonal close-packed (hcp)^24−26^ kernels, the combination of phosphine and thiolate/selenolate ligands is typically required for assemblies of icosahedral Au_13_ building blocks into multiunit superstructures, often referred to as “clusters of clusters”.^27−29^ For instance, bi-icosahedral Au_25_^30^ and tri-icosahedral Au_37_^31^ nanoclusters in a rod shape have been successfully prepared through the use of triphenylphosphine and aliphatic thiolates. In addition, triphenylphosphine and benzeneselenolate can co-stabilize a ring-like Au_60_ nanocluster based on five vertex-sharing icosahedra.^32^ Although the three-dimensional (3D) assembly of 13-atom centered icosahedral cluster units was predicted in 1987,^29^ finding suitable ligands to access the multi-icosahedral gold nanoclusters with 3D assembling structures remains a significant challenge to date.

**Scheme 1:**
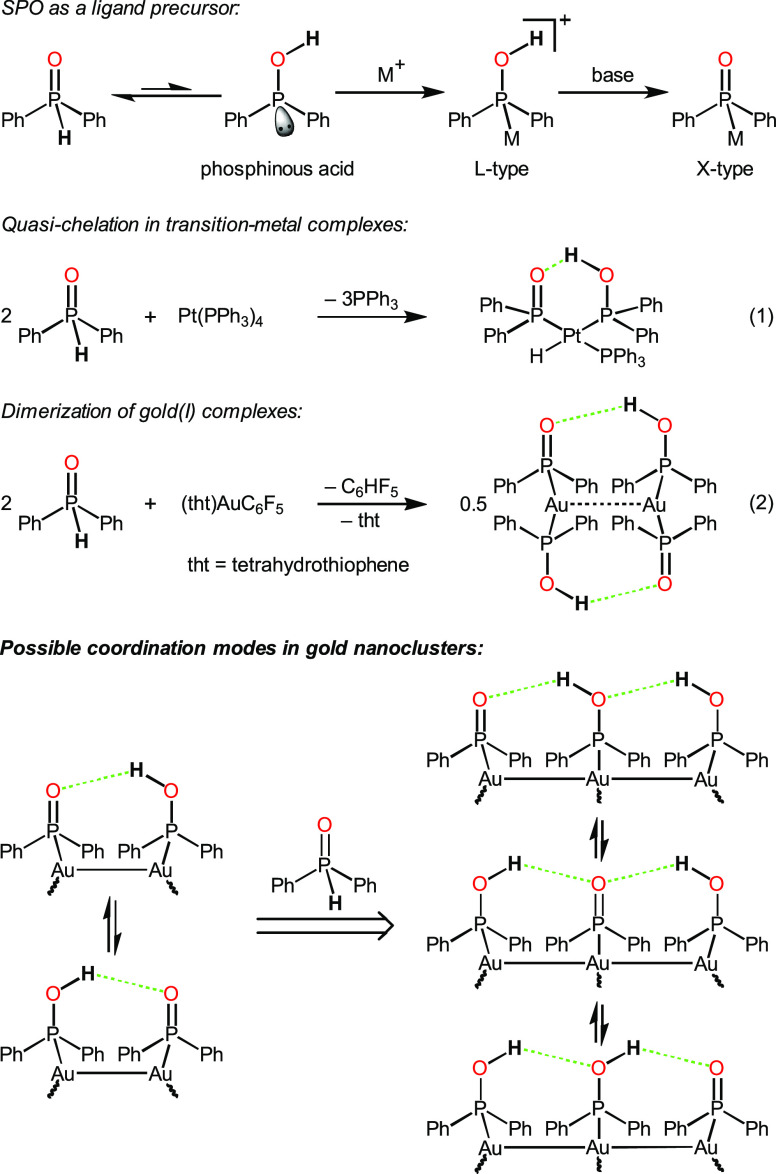
Construction of Phosphinous Acid–Phosphinito Transition-Metal Complexes and Nanoclusters Based on Hydrogen Bonding

In order to further diversify the kernel structures of coinage metal nanoclusters in dual ligand systems, we turn to secondary phosphine oxides (SPOs) as a replacement for phosphines.^33−36^ It is well-studied that SPOs are in a tautomeric equilibrium with the trivalent phosphinous acids which can strongly bind to soft transition metals via the phosphorus atom (Scheme 1).^37−39^ In this scenario, phosphinous acids bearing a P–O single bond serve as neutral L-type ligands, with a coordination mode comparable to those of phosphines.^40,41^ When phosphinous acids are deprotonated with external Brønsted bases, the newly generated phosphinito ligands become anionic X-type, having predominantly double bond characters in P=O.^36^ In sharp contrast to commonly used thiolate ligands, the phosphorus center on anionic phosphinito ligands only interacts with one gold atom at a time, which may have a significant effect on the packing of icosahedral Au_13_ units. Moreover, Roundhill and co-workers revealed that the intramolecular hydrogen bond between the phosphinous acid and phosphinito ligands (P–O–H···O=P) offered additional stability to the platinum complex through a quasi-chelation effect (Scheme 1, eq 1).^42^ This type of P–O–H···O=P bonding also triggered dimerization of a neutral gold(I) complex, along with the contributions from Au···Au interactions (Scheme 1, eq 2).^43^ Given that the gold atoms in the kernel’s outer shell are likely to have only one coordination site available, we envision that a set of phosphinous acid and phosphinito ligands would protect two to three gold atoms in close proximity and provide extra stabilization to the nanoclusters through hydrogen-bonding interactions (Scheme 1). The protons may shuttle between the neighboring oxygen atoms in intramolecular protonation–deprotonation equilibria.^39,43,44^ The whole collection of the phosphorus-based protecting ligands can be described as a phosphinous acid–phosphinito (PAP) ligand set.

Herein, we report the synthesis and total structure determination of the first gold nanocluster co-stabilized by diphenylphosphinous acid (HOPPh_2_), anionic diphenylphosphinito ligand (OPPh_2_), and 4-*tert*-butylbenzenethiolate (TBBT): Au_52_(HOPPh_2_)_8_(OPPh_2_)_4_(TBBT)_16_ (denoted as **Au_52_-PAP**). The kernel of **Au_52_-PAP** contains four icosahedral Au_13_ units that are linked together in an unexpected 3D assembly. **Au_52_-PAP** offers remarkable antioxidant properties and multiple activation sites for oxygen binding, which enables its use in electrochemical oxygen reduction reactions (ORR) with great reactivity and durability. According to computational studies, the most active gold centers for the ORR are those coordinated by the PAP ligands.

## Results and Discussion

### Synthesis and Structural Analysis of Au_52_(HOPPh_2_)_8_(OPPh_2_)_4_(TBBT)_16_

A modified two-step synthesis procedure was utilized to prepare **Au_52_-PAP** containing both sulfur- and phosphorus-donor ligands.^45,46^ To begin, we made Au_*n*_(HOPPh_2_)_*x*_(OPPh_2_)_*y*_(TBBT)_*z*_ clusters of various sizes by reducing chloroauric acid with NaBH_4_ in the presence of diphenylphosphine oxide and 4-*tert*-butylbenzenethiol (TBBTH). In the second step, the gold precursors and excess TBBTH were dissolved in toluene, and a small amount of water was added to separate the reaction mixture into two phases. After a 6 h size-aging process at 75 °C, the phosphinous acid–phosphinito gold nanocluster (**Au_52_-PAP**) formed, which was subsequently purified using preparative thin-layer chromatography. The overall yield of **Au_52_-PAP** was 2%, based on chloroauric acid utilized in the first step.

**Figure 1:**
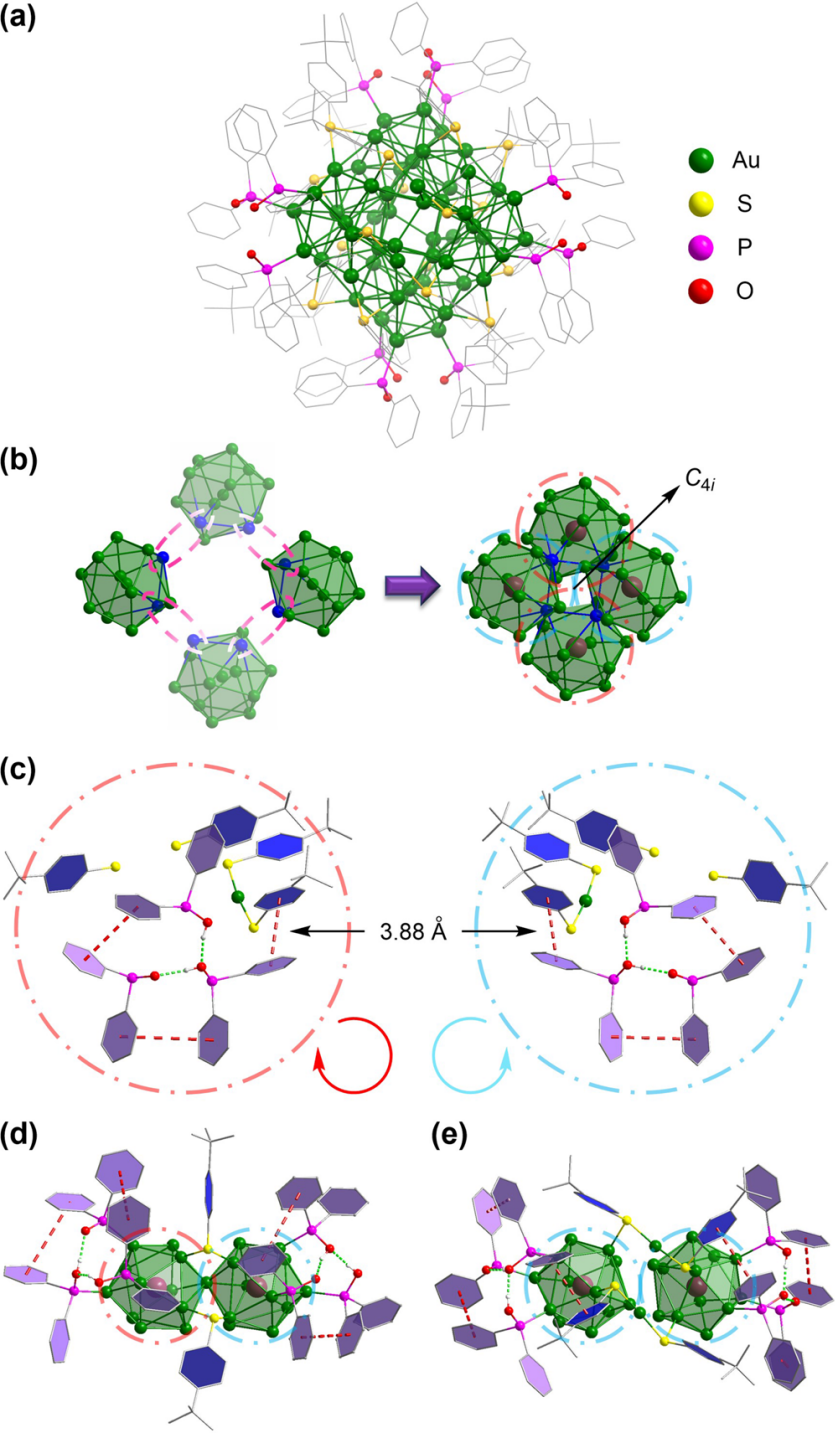
(a) Total structure of the Au_52_(HOPPh_2_)_8_(OPPh_2_)_4_(TBBT)_16_ nanocluster (color labels: Au, green; S, yellow; P, magenta; O, red; C, gray). All hydrogen atoms are omitted for clarity. (b) Four icosahedral Au_13_ units form an Au_48_ kernel by sharing four vertex gold atoms. The shared vertex gold atoms are highlighted in blue. The brown balls represent the centers of the Au_13_ units. (c) Red and blue circles represent the clockwise and anticlockwise orientations from the aryl group of the TBBT ligand to the phenyl groups of the PAP ligands, respectively. The red dashed lines depict the π–π interactions between the PAP ligands and the TBBT motif of the −SR–Au–SR– staple. The green dashed lines depict the hydrogen-bonding interactions within the PAP ligands. (d) Two TBBT ligands connect two vertex-sharing icosahedral Au_13_ units. (e) Two −SR–Au–SR– staples connect two separated icosahedral Au_13_ units.

Single-crystal X-ray diffraction revealed that the crystals of **Au_52_-PAP** belong to the triclinic *P*-1 space group. The total structure of **Au_52_-PAP** comprises an Au_48_ kernel and a protective shell containing four monomeric gold–thiolate (−SR–Au–SR−) staples (SR = TBBT), eight bridging TBBT ligands, and four sets of hydrogen-bonded PAP (Ph_2_P=O···HOPPh_2_···HOPPh_2_) ligands (Figure 1a). The Au_48_ kernel can be viewed as a 3D assembly of four icosahedral Au_13_ units; each adjacent two units share a vertex gold atom in a cyclic fashion (Figure 1b). As with the carbon atoms in the cyclobutane ring, these four icosahedra, which share four vertices in total, are not on the same plane, giving a 3D “puckered” shape with a nuclearity of 48 in the kernel (Figure S3). In each icosahedral unit, the lengths of the Au_c_–Au_p_ and Au_p_–Au_p_ bonds are approximately 2.80 and 2.95 Å (Au_c_ and Au_p_ represent the central and peripheral gold atoms of the Au_13_ icosahedra, respectively). The average distance between the gold atom of the staple and the peripheral gold atom of the icosahedral unit is 3.12 Å (Figure S4). The structure of the entire kernel, as well as the distribution patterns of the ligands in the protective shell, possesses a unique *C*_4*i*_ symmetry. According to Teo’s prediction,^29,47^ however, each Au_13_ unit should share three vertex gold atoms with the other three, resulting in a tetrahedral array of the four centered icosahedra with a nuclearity of 46 (Figure S5). In the predicted structure, there are three *S*_4_ axes orthogonal to one another. Regarding the formation of the unexpected Au_48_ kernel in **Au_52_-PAP**, we believe that the synergistic stabilization effects from both the PAP ligands and the gold–thiolate staples are of great importance.

When the centers of the four icosahedral units are connected, a distorted tetrahedron with four short edges (average length of 5.67 Å) and two long edges (average length of 6.48 Å) is produced (Figure S6). The long edges are perpendicular to each other, with their centers along the *C*_4*i*_ axis. The ligand arrangements are equal on the unconnected icosahedral Au_13_ units but opposite on the neighboring ones, which is in accordance with the *C*_4*i*_ symmetry of the whole cluster. To be more specific, from the TBBT motifs of the staple to the phenyl groups of the PAP ligands, these aryl rings are oriented clockwise on two of the icosahedral units and anticlockwise on the other two (Figure 1c). Two phosphinous acids and one phosphinito ligand are connected through two strong hydrogen bonds with an average O···HO distance of 1.68 Å,^48^ which is supported by the IR results (Figures S7 and S8).^43^ The average length of the P–OH bonds in the PAP is 1.59 Å, almost identical to that in [Pd_2_(HOP*t*-Bu_2_)_2_Cl_4_].^41^ The average PO distance of the phosphinito ligands is 1.55 Å, which is about 0.05 Å longer than the P=O double bonds in free SPOs^49,50^ and [Au(OPPh_2_)_2_]^−^;^51^ this difference could be attributed to the intramolecular hydrogen bond between the phosphinous acid and the phosphinito ligand.^43,44^ The phosphinito ligand also interacts with the two phosphinous acids nearby via π–π stacking. More importantly, one of the phosphinous acids in the PAP has a strong π–π stacking interaction with one TBBT motif in the −SR–Au–SR– staple, giving an average distance of 3.88 Å.^52^ While a pair of the bridging TBBT ligands connects a pair of the vertex-sharing icosahedra (Figure 1d), a pair of −SR–Au–SR– staples binds a pair of the isolated icosahedra (Figure 1e). The π–π stacking interactions between the −SR–Au–SR– staples and the PAP ligands of the unconnected icosahedral Au_13_ units help maintain the kernel structure of **Au_52_-PAP**, which just contains four shared vertex gold atoms. The well-organized distribution of the PAP and TBBT ligands, as well as their synergistic stabilization effects, results in extraordinarily high stability for **Au_52_-PAP**, paving the way for the comprehensive nuclear magnetic resonance (NMR) studies and possible applications in electrochemical catalysis.

### NMR and MS Studies

**Figure 2:**
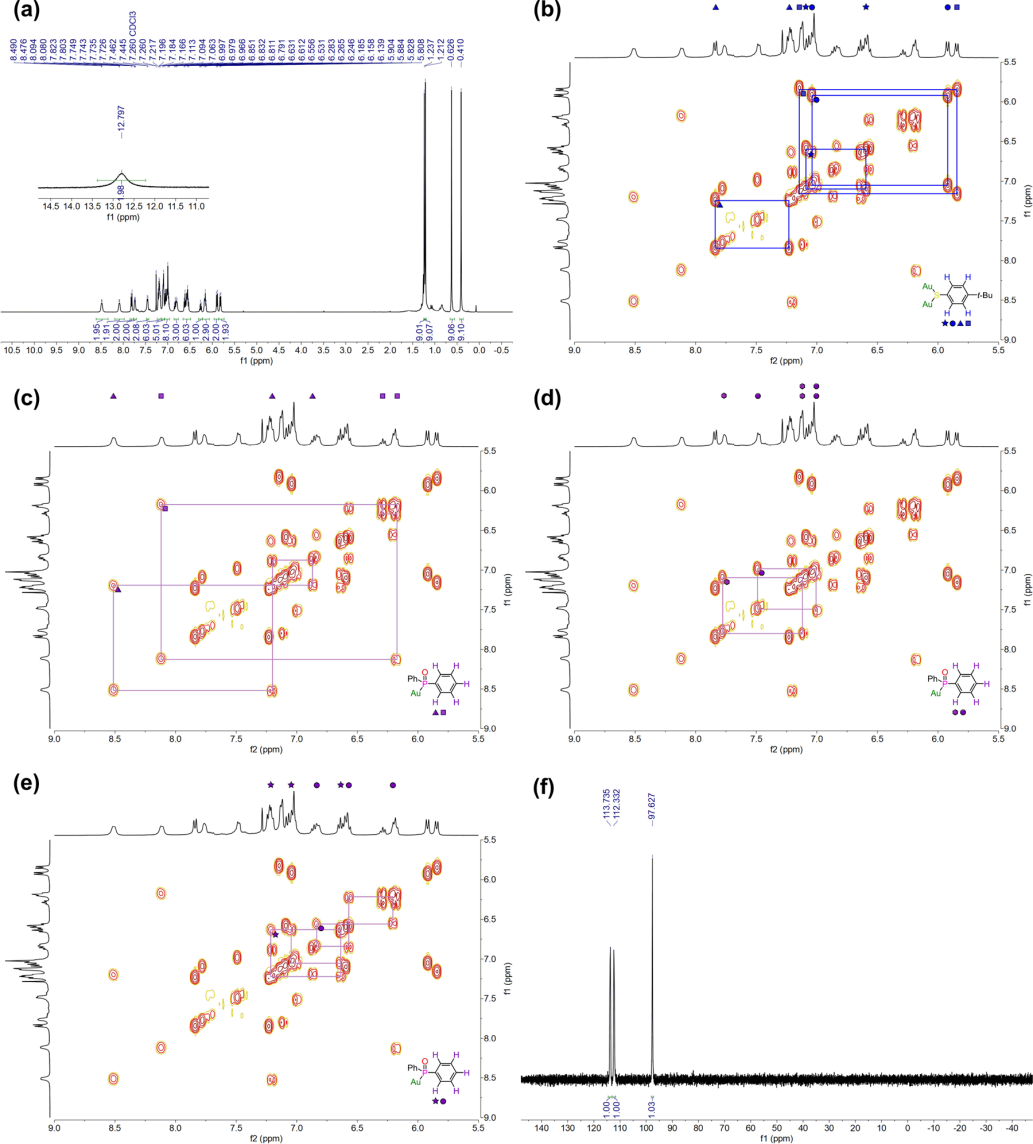
(a) ^1^H NMR spectrum of **Au_52_-PAP**. (b–e) ^1^H–^1^H COSY spectrum depicting the correlations between (b) protons in TBBT ligands and (c–e) protons in PAP ligands with different chemical environments. (f) ^31^P NMR spectrum of **Au_52_-PAP**.

The surface of **Au_52_-PAP** is protected by 16 sulfur-donor ligands in four coordination environments and 12 phosphorus-donor ligands in three coordination environments, which is further supported by NMR analysis shown in Figure 2. The four −SR–Au–SR– staples, attached to two sets of Au_13_ icosahedra that share no vertices, can be divided into two groups, one of which is shown in Figure 1e. Due to the *C*_2_ symmetry of each group, the TBBT ligands decorated on the staples provide two sets of distinguishable signals in the ^1^H NMR and the corresponding ^1^H–^1^H COSY spectra (Figure 2a,b). The remaining eight TBBT ligands are divided into four groups, each of which is directly connected to a pair of Au_13_ icosahedra with a shared vertex (Figure 1d), responding to the other two sets of TBBT signals in Figure 2a,b. The PAP ligands are distributed evenly among the icosahedral Au_13_ units; the two phenyl groups on each phosphorus-donor ligand have distinct conformations and interact differently with the kernel and other ligands. Thus, six sets of aromatic signals together with three phosphorus resonances can be clearly identified in the ^1^H–^1^H COSY and ^31^P NMR spectra (Figure 2c–f). Since the P–O bonds in phosphinous acids are protonated, their ^31^P NMR peaks are more downfield than that of the phosphinito ligand (97.6 ppm).^51^ The presence of the active protons at 12.80 ppm in the ^1^H NMR spectrum indicates that there are eight phosphinous acid ligands in the nanocluster (Figure 2a).

**Figure 3:**
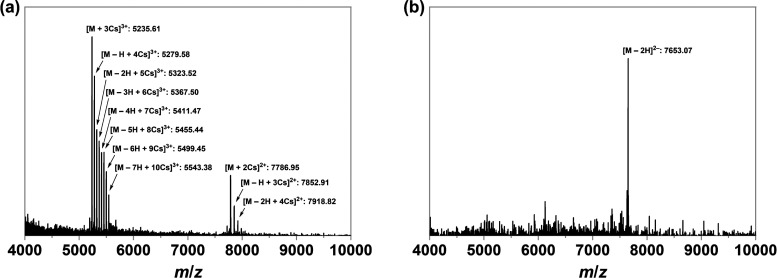
(a) ESI-MS of **Au_52_-PAP** in a positive-ion mode. (b) ESI-MS of **Au_52_-PAP** in a negative-ion mode.

The molecular formula of the targeted nanocluster was determined using electrospray ionization mass spectrometry (ESI-MS) in both positive- and negative-ion modes (Figure 3).^53,54^ Two sets of trication and dication signals as well as one major dianion signal were detected over the mass-to-charge ratio (*m*/*z*) range of 4000–10,000. The peaks at *m*/*z* 5235.61, 5279.58, 5323.52, 5367.50, 5411.47, 5455.44, 5499.45, and 5543.38 correspond to [Au_52_(HOPPh_2_)_8_(OPPh_2_)_4_(TBBT)_16_ – *n*H + (3 + *n*)Cs]^3+^ (*n* = 0–7). The peaks at *m*/*z* 7786.95, 7852.91, and 7918.82 correspond to [Au_52_(HOPPh_2_)_8_(OPPh_2_)_4_(TBBT)_16_ – *n*H + (2 + *n*)Cs]^2+^ (*n* = 0–2). The peaks at *m*/*z* 7653.07 is associated with [Au_52_(HOPPh_2_)_8_(OPPh_2_)_4_(TBBT)_16_ – 2H]^2–^. Their isotropic distributions closely matched those of the simulations (Figure S9). The easy replacement of the protons with cesium ions, as well as the detection of the anionic base form in the negative-ion mode of ESI-MS, strongly suggests the presence of multiple phosphinous acids in the protective shell of **Au_52_-PAP**. The elemental composition of **Au_52_-PAP** was further investigated by X-ray photoelectron spectroscopy. All the expected elements (i.e., Au, O, S, P, and C) were observed in the survey spectrum of **Au_52_-PAP** (Figure S10). The high-resolution Au4f spectrum revealed a Au 4f_7/2_ peak at 84.26 eV and a Au 4f_5/2_ peak at 87.92 eV, indicating the presence of Au(0) in this gold nanocluster (Figure S11).^55^ According to transmission electron microscopy, the ultrasmall **Au_52_-PAP** nanoclusters had an average particle size of 1.42 nm (Figure S12).

### UV–vis–NIR Absorption and Valence Electron Determination

**Figure 4:**
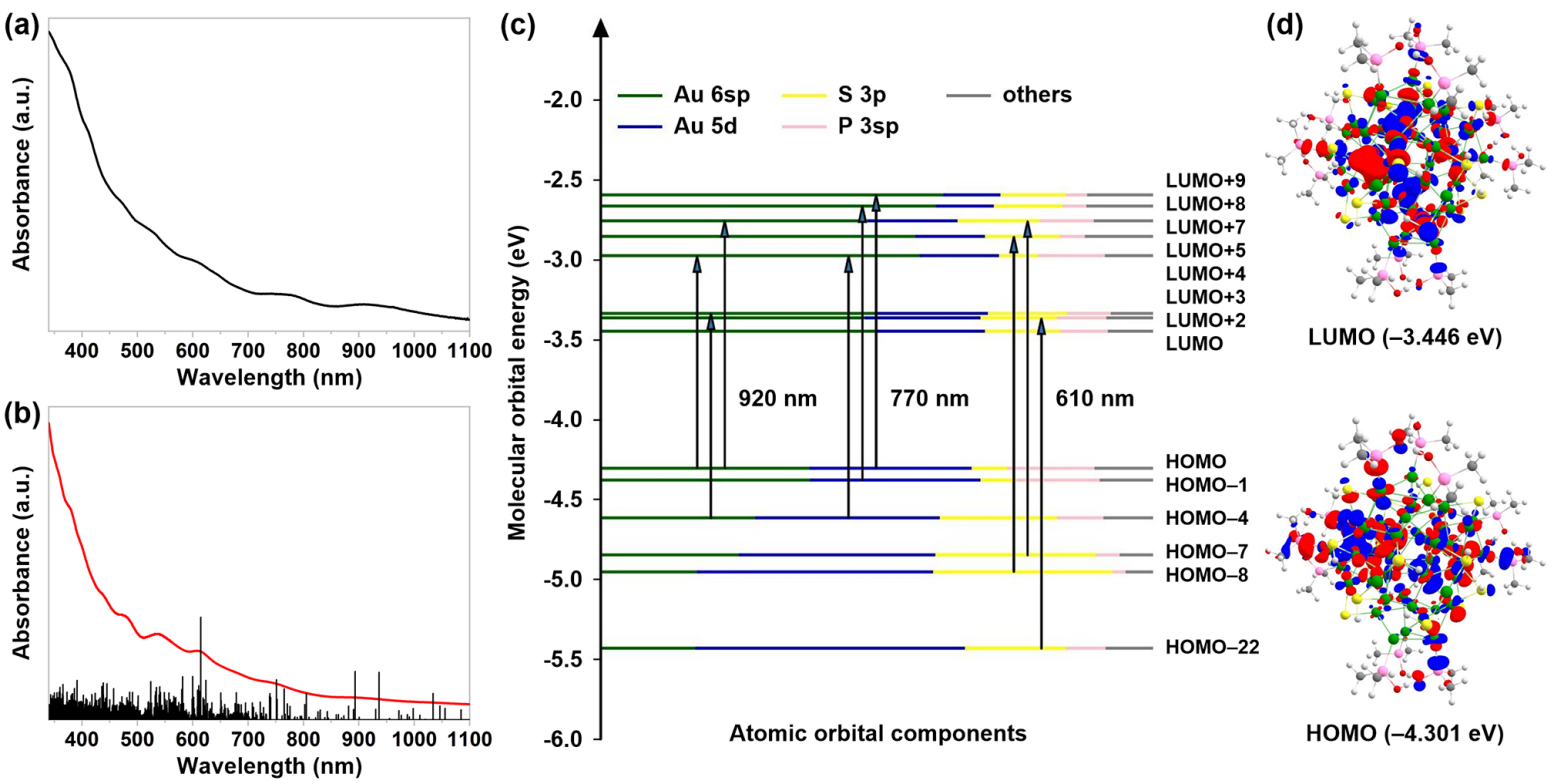
(a) Experimental and (b) simulated UV–vis–NIR absorption spectra of **Au_52_-PAP** in CH_2_Cl_2_. (c) K–S molecular orbital energy level diagram of **Au_52_-PAP**. (d) HOMO and LUMO distributions of **Au_52_-PAP**.

The UV–vis–NIR spectrum of **Au_52_-PAP** displays six absorption bands centered at 380 (ε_1_: 1.55 × 10^5^ M^–1^ cm^–1^), 470 (ε_2_: 0.78 × 10^5^ M^–1^ cm^–1^), 530 (ε_3_: 0.58 × 10^5^ M^–1^ cm^–1^), 610 (ε_4_: 0.42 × 10^5^ M^–1^ cm^–1^), 770 (ε_5_: 0.21 × 10^5^ M^–1^ cm^–1^), and 920 nm (Figure 4a). To assign the signature of the absorption spectrum of **Au_52_-PAP**, time-dependent density functional theory calculations were performed based on its single-crystal structure.

The calculated absorption spectrum (Figure 4b) is largely comparable with the experimental result. As illustrated in Figure 4c, the theoretical analysis of the atomic orbital components in the frontier Kohn–Sham (K–S) molecular orbitals of **Au_52_-PAP** attributes the sharp absorption peak at 610 nm to the HOMO–8 → LUMO+5, HOMO–7 → LUMO+7, and HOMO–22 → LUMO+2 electronic transitions, the absorption peak at 770 nm to the HOMO–4 → LUMO+4, HOMO–1 → LUMO+8, and HOMO → LUMO+9 electronic transitions, and the absorption peak at 920 nm to the HOMO → LUMO+4, HOMO–4 → LUMO+3, and HOMO → LUMO+7 electronic transitions. From the low-energy HOMOs (e.g., HOMO–22) to the high-energy LUMOs (e.g., LUMO+9) in the K–S orbitals of **Au_52_-PAP**, the gold atoms’ major contributions shift from their Au 5d to Au 6sp atomic orbitals. The electronic transitions responsible for the absorption peak at 610 nm can be classified as the interband (d → sp) transitions, whereas those responsible for the absorption peaks at 770 and 920 nm involve the redistribution of the Au 5d and Au 6sp atomic orbital components. The HOMO, LUMO, and remaining molecular orbital distributions of **Au_52_-PAP** are shown in Figures 4d and S17, respectively, and the HOMO–LUMO gap is estimated to be 0.86 eV, which is much smaller than that (1.44 eV) of Au_52_(TBBT)_32_ (denoted as **Au_52_**) in a fcc structure (Figure S18).^21^

The number of total valence electrons for **Au_52_-PAP** was calculated to be 32 (52 – 4 – 16). In order to further analyze the kernel of the ligand-protected nanocluster, the following protocols were utilized to determine the formal oxidation states of the gold atoms (Figure S13).^56^ When two gold atoms are bridged by a thiolate ligand or a −SR–Au–SR– staple, their formal oxidation state is +0.5 (with 0.5 valence electron). The gold atom coordinated by an anionic phosphinito ligand has a formal oxidation state of +1 (with 0 valance electron), whereas the gold atom coordinated by a phosphinous acid has a formal oxidation state of 0 (with 1 valence electron). The kernel of **Au_52_-PAP** can be separated into four icosahedral Au_13_ units in the I_47_ valence state, according to a grand unified model.^56^ These superatomic building units all have a noble-gas-like 8-electron configuration (1S^2^1P^6^).^57,58^ If there were only phosphinito and thiolate ligands in the neutral cluster, the Au_13_ unit would adopt an unstable 6-electron configuration (Figure S14). Determining the valence electrons of the Au_13_ icosahedra provides additional support for the presence of L-type phosphinous acid ligands in **Au_52_-PAP**. Notably, when exposed to hydrogen peroxide, **Au_52_-PAP** with the electronically closed shell superatoms demonstrated considerably higher antioxidant capacity than **Au_52_** (Figure S15).

### Electrocatalytic ORR with **Au_52_-PAP**

**Figure 5:**
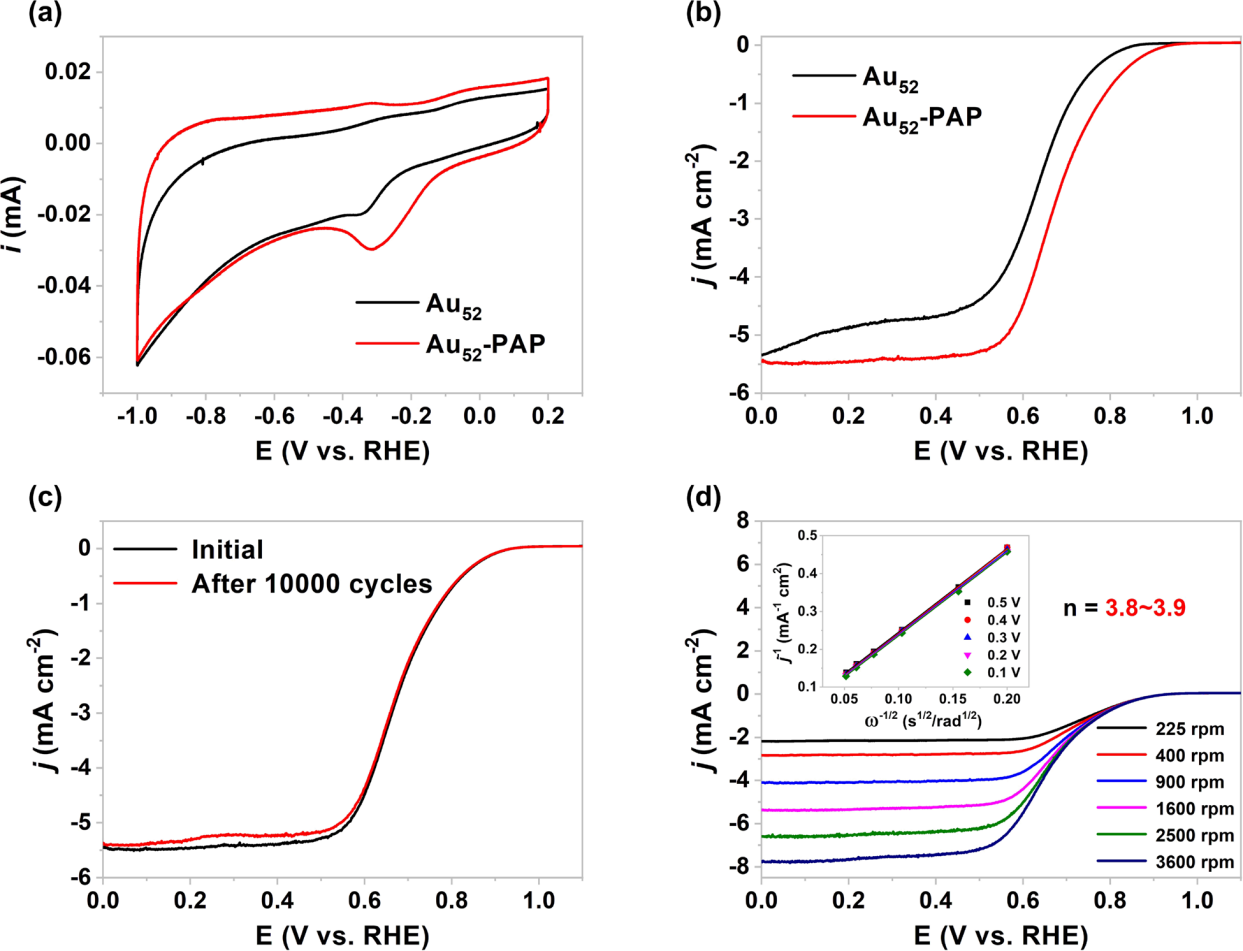
(a) CV curves of **Au_52_-PAP** and **Au_52_** in aqueous KOH (0.1 M) at a scan rate of 50 mV/s. (b) ORR polarization curves of **Au_52_-PAP** and **Au_52_** in an O_2_-saturated aqueous solution of KOH (0.1 M) at a scan rate of 10 mV/s and a rotation speed of 1600 rpm. (c) ORR polarization curves of **Au_52_-PAP** before and after 10,000 cycles. (d) ORR polarization curves of **Au_52_-PAP** at different rotation speeds. Inset: K–L plots at different potentials.

On account of the great chemical stability of **Au_52_-PAP**, we used the ORR as a model reaction to survey its electrocatalytic performance, while **Au_52_** protected by the same thiolate ligand but in a different kernel-packing mode served as a control. As shown in Figure 5a, the cyclic voltammogram (CV) curves of **Au_52_-PAP** and **Au_52_** in an aqueous KOH electrolyte exhibit a clear reduction peak toward the negative scan direction, which could be ascribed to the reduction of Au–O species.^59,60^ At the same scan rate, the cathodic peak potential of **Au_52_-PAP** is more positive than that of **Au_52_**, which indicates a weaker bonding between the hydroxyl group, a key intermediate in the ORR, and the catalytically active gold center on **Au_52_-PAP**.^61^ Importantly, the polarization curves for ORR reveal that **Au_52_-PAP** has a more positive onset potential [(0.90 V; all the potentials are referenced to a reversible hydrogen electrode (RHE)] than **Au_52_** (0.82 V) (Figure 5b). In addition, the half-wave potential of **Au_52_-PAP** (0.69 V) has an obviously positive shift when compared to that of **Au_52_** (0.63 V). The relatively positive onset potential and half-wave potential indicate that **Au_52_-PAP** could catalyze the reduction of oxygen molecules more efficiently.^62^ The current density of **Au_52_-PAP** is also higher throughout the entire potential range. It is worth noting that the specific activity of **Au_52_-PAP** at 0.8 V, obtained by normalizing the current with the area of the electrode (0.20 cm^2^), reaches −0.67 mA/cm^2^, whereas the specific activity of **Au_52_** at 0.8 V is only −0.17 mA/cm^2^. The mass activity of **Au_52_-PAP** at 0.8 V, obtained by normalizing the current with the mass of the gold nanocluster on the electrode, is 0.11 A/mg, about 3.5 times higher than that of **Au_52_**. The superior specific activity and mass activity of **Au_52_-PAP** confirm that it is a better electrocatalyst for the ORR in comparison to the previously reported **Au_52_**. Moreover, **Au_52_-PAP** has remarkable electrocatalytic durability in the ORR processes. As illustrated in Figure 5c, after completing an accelerated durability test for 10,000 cycles, its half-wave potential and the current density at 0.8 V remained nearly unchanged. The absence of agglomerated particles and the constant average size following the accelerated durability test suggested that **Au_52_-PAP**’s structure was essentially stable during the electrocatalytic ORR (Figure S16). To investigate the ORR mechanism with **Au_52_-PAP**, we measured the polarization curves at various rotation speeds (Figure 5d). The almost parallel Koutecký–Levich (K–L) plots inserted in Figure 5d demonstrate the first-order reaction kinetics toward the oxygen dissolved in the electrolyte. Based on the K–L equation, the electron transfer number (*n*) is determined to be 3.8 to 3.9 over the entire potential range, indicating that the four-electron transfer pathway is dominant for the ORR on **Au_52_-PAP**.

**Figure 6:**
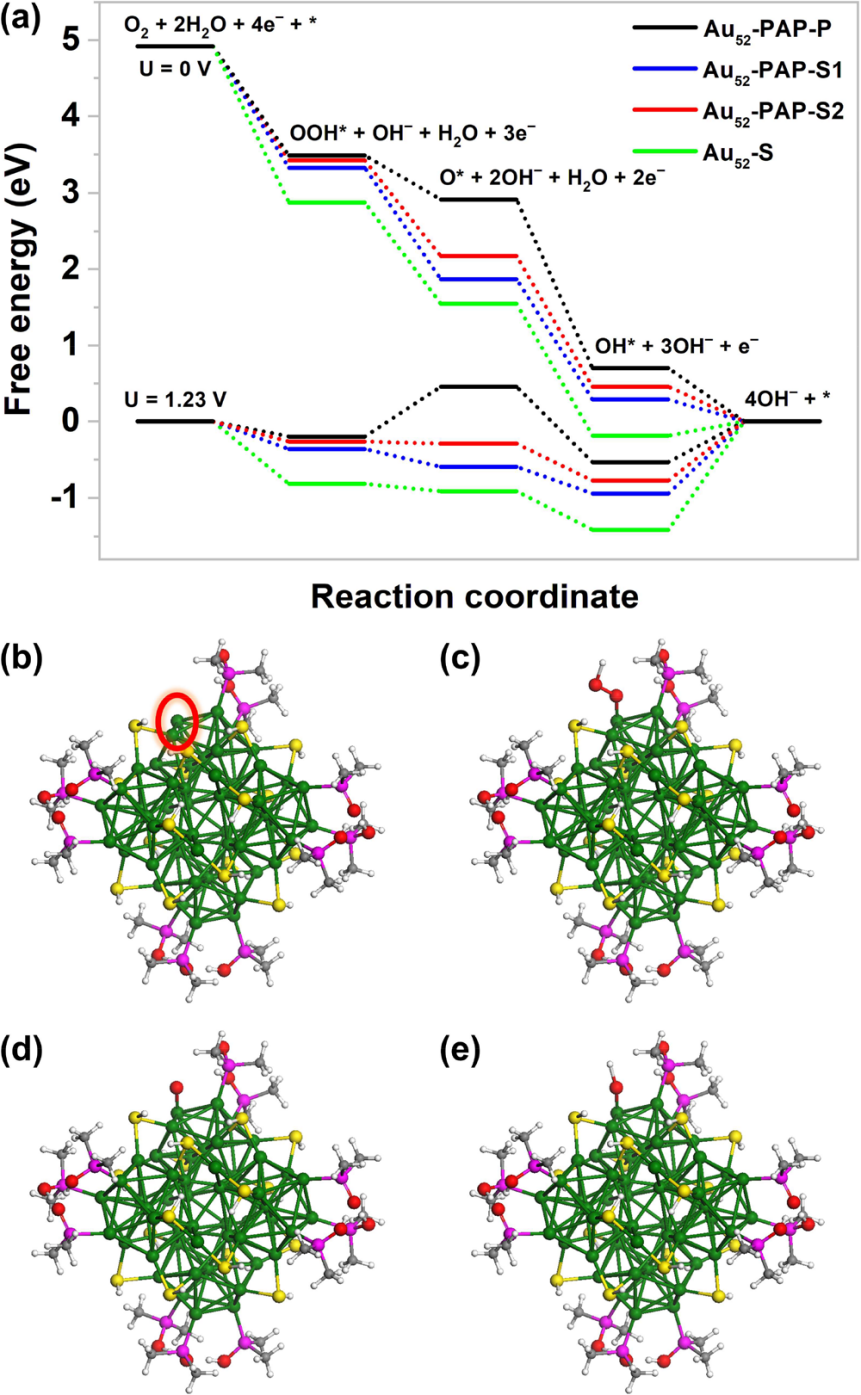
(a) Free energy diagram for ORR pathways on the active sites of **Au_52_-PAP** and **Au_52_** in alkaline media (pH = 13). (b) Removal of the phosphinito ligand for exposing the highly active gold center on **Au_52_-PAP** to provide activated gold catalyst **Au_52_-PAP-P**. (c) Adsorption of OOH* to **Au_52_-PAP-P**. (d) Adsorption of O* to **Au_52_-PAP-P**. (e) Adsorption of OH* to **Au_52_-PAP-P**. Color labels: green, Au; yellow, S; magenta, P; red, O; gray, C; white, H.

To gain a better understanding of the ORR mechanisms, we conducted comprehensive density functional theory (DFT) calculations to determine the origin of the difference in electrocatalytic performances between **Au_52_-PAP** and **Au_52_**, as well as the most active sites on **Au_52_-PAP** (Figure 6). Considering that the active gold centers on the nanoclusters would become unbonded during the course of the electrochemical catalysis,^63,64^ we first removed the phosphinito ligand (i.e., OPMe_2_) and the thiolate ligands (i.e., SH) of the −SR–Au–SR– staple from **Au_52_-PAP** to produce the corresponding activated gold catalysts (**Au_52_-PAP-P**, **Au_52_-PAP-S1**, and **Au_52_-PAP-S2**, respectively; see Figures 6b, S19a, and S20a). As for the ordinary gold nanocluster (**Au_52_**), the thiolate ligand on a gold atom of the (111) surface was removed to give **Au_52_-S** (Figure S21a). The free energy diagram of the ORR processes with these activated catalysts at 0 and 1.23 V is shown in Figure 6a. When no external potential is applied (*U* = 0 V), the free energy of the ORR intermediates decreases step by step. While *U* = 1.23 V, the energy gaps are considerably narrowed in most of the reduction steps. As with **Au_52_-S**, DFT calculations suggest that the OOH*, O*, and OH* intermediates of **Au_52_-PAP-S1** and **Au_52_-PAP-S2** interact strongly with two adjacent gold atoms (Figures S19–S21), resulting in a decrease in the free energy for the OOH* and O* formation but a significant increase in the free energy for the ultimate OH* desorption. Therefore, the rate-determining step (RDS) for the ORR is the desorption of OH* from the active gold centers of **Au_52_-PAP-S1**, **Au_52_-PAP-S2**, and **Au_52_-S**, with free energies raised by 0.95, 0.78, and 1.40 eV, respectively (Figure 6a). According to the density of states of the activated gold catalysts, the d-band center of **Au_52_-S** is the most upshifted (Figure S22), indicating the strongest interaction between its active gold centers and the adsorbates (i.e., OOH*, O*, and OH*); thus, **Au_52_** is not as effective as **Au_52_-PAP** in promoting the ORR processes. In sharp contrast, the OOH*, O*, and OH* intermediates can only bind to a single gold atom upon removal of the phosphinito ligand from **Au_52_-PAP**. While this binding mode further reduces the energy gap in the OH* desorption step to 0.53 eV, the loss of the stabilizing effect from a second gold atom leads to a dramatic increase in the free energy for the O* formation on **Au_52_-PAP-P** (0.65 eV). As a result, the O* formation step becomes the RDS in the ORR pathway involving **Au_52_-PAP-P**. Due to the smallest energy gap in the RDS, **Au_52_-PAP-P** could catalyze the reduction of oxygen molecules most efficiently under the electrochemical conditions.

## Conclusions

In summary, we have successfully synthesized the first phosphinous acid–phosphinito gold nanocluster **Au_52_-PAP**, the kernel of which is built via an unexpected 3D assembly of four vertex-sharing icosahedral Au_13_ units. As opposed to the predicted *T_d_* symmetry, **Au_52_-PAP** possesses an unusual *C*_4*i*_ symmetry, with just four shared vertex gold atoms. The Au_13_ icosahedra that share no vertices are stabilized by the hydrogen-bonding interactions within the PAP ligand sets and the π–π stacking interactions between the PAP ligands and the −SR–Au–SR– staples. The incorporation of the PAP ligands into the protective shell of **Au_52_-PAP** not only enhances its antioxidant capacity but also generates highly active sites on the gold nanocluster for promoting the ORR processes. Notably, the presence of phosphinous acids in the PAP ligand sets of **Au_52_-PAP** is confirmed by the NMR, IR, and MS analyses, as well as the closed-shell electronic configuration of the Au_13_ superatomic atoms, which is essential for the future development of gold nanoclusters based on SPO ligand precursors.

## Methods

### Synthesis of **Au_52_-PAP**

In a 100 mL three-neck flask, tetraoctylammonium bromide (400 mg, 0.732 mmol) and HAuCl_4_·4H_2_O (200 mg, 0.486 mmol) were initially dissolved in tetrahydrofuran (15 mL). After stirring at 350 rpm for 5 min, O=PHPh_2_ (100 mg, 0.495 mmol) was added; the color of the solution changed from deep red to yellow. TBBTH (400 mg, 2.41 mmol) was added afterward. After 10 min, a freshly prepared 3 mL cold aqueous solution of NaBH_4_ (260 mg, 6.88 mmol) was added instantaneously. Then, 40% sulfuric acid (2.0 mL) was added to the solution. The gold nanoclusters were allowed to grow for 5 min. The multi-sized Au_*n*_(HOPPh_2_)_*x*_(OPPh_2_)_*y*_(TBBT)_*z*_ cluster precursors were obtained upon addition of excess methanol and collected by centrifugation. The second step involved a two-phase reaction. In detail, the obtained precursors and excess TBBTH (300 mg, 1.81 mmol) were dissolved in toluene (10 mL), and water (20 mL) was added to separate the reaction mixture into two phases. The resulting solution was heated at 75 °C for 6 h. Black solids were obtained after adding excess amounts of methanol. The residue was centrifuged and washed with methanol five times to remove free thiols and other byproducts. The nanocluster was purified by preparative thin-layer chromatography (PTLC). The desired product on the PTLC plates was collected and extracted with dichloromethane three times. The combined organic layers were removed in a vacuum to give **Au_52_-PAP**.

### Electrocatalytic ORR

Electrochemical measurements were conducted in a standard three-electrode cell which was connected to a Bio-logic VMP3 potentiostat. A leak-free Ag/AgCl electrode and a platinum mesh (1 × 1 cm^2^) attached to a platinum wire were used as the reference and counter electrodes, respectively.

The working electrode was prepared as follows: carbon-supported nanoclusters (5 mg) were dispersed into a mixture containing ethanol (1 mL) and 5 wt % Nafion solution (0.050 mL), followed by an ultrasonic treatment for 30 min to form a uniform catalyst ink. Then, 5 μL of the ink was dropped onto a glassy carbon disk electrode (5 mm diameter). Subsequently, the glassy carbon electrode was dried in a warm air stream at 70 °C for 1 h.

The linear sweep voltammetry curves of the **Au_52_-PAP** and **Au_52_** nanoclusters for the ORR at room temperature were obtained by a rotating disk electrode test, which was carried out in an O_2_-saturated 0.1 M KOH solution with a scan rate of 10 mV/s at different rotation rates. The long-term durability tests were performed by continuously sweeping potential cycles in the potential range of 0.6–1.0 V vs RHE with an accelerated sweep rate of 100 mV/s in an O_2_-saturated 0.1 M KOH solution at room temperature.

## Supporting Information Available

Detailed synthetic procedures, characterization data, electrochemical measurements, and computational details (PDF)

## Accession Codes

CCDC 2177432 contains the supplementary crystallographic data for this paper. These data can be obtained free of charge via www.ccdc.cam.ac.uk/data_request/cif, or by emailing data_request@ccdc.cam.ac.uk, or by contacting The Cambridge Crystallographic Data Centre, 12 Union Road, Cambridge CB2 1EZ, UK; fax: +441223 336033.
