# Supplementary material for: Phosphinous Acid–Phosphinito Tetra-Icosahedral Au52 Nanoclusters for Electrocatalytic Oxygen Reduction
Source: JACS Au. 2022 Nov 3;2(11):2617–26. doi: 10.1021/jacsau.2c00517 (PMC9709937; doi:10.1021/jacsau.2c00517)
Supplement: Supplementary file 1 — au2c00517_si_001.pdf [file au2c00517_si_001.pdf]

# *Supporting Information*

# Phosphinous Acid–Phosphinito Tetra-icosahedral $Au_{52}$ Nanocluster for Electrocatalytic Oxygen Reduction

Shengli Zhuang,[1,2†] Dong Chen,[3†] Wai-Pan NG,[1†] Dongyi Liu,[1] Li-Juan Liu,[1] Meng-Ying Sun,[1] Tehseen Nawaz,[1] Xia Wu,[1] Yao Zhang,[1] Zekun Li,[1] Yong-Liang Huang,[4] Jun YANG,[1]* Jun Yang,[3]* and Jian He[1,2]*

[1]Department of Chemistry, The University of Hong Kong, Pokfulam Road, Hong Kong, P. R. China

[2]State Key Laboratory of Synthetic Chemistry, The University of Hong Kong, Pokfulam Road, Hong Kong, P. R. China

[3]State Key Laboratory of Multiphase Complex Systems, Institute of Process Engineering, Chinese Academy of Sciences, Beijing 100190, P. R. China

[4]Department of Medicinal Chemistry, Shantou University Medical College, Shantou, Guangdong 515041, P. R. China

[†]These authors contributed equally.

Email: jianhe@hku.hk
Email: jyang@ipe.ac.cn
Email: juny@hku.hk

## Table of Contents

1. Experimental details S–2

2. Characterization S–2

3. Electrochemical measurements S–13

4. Density functional theory calculations S–15

5. References S–19

## 1. Experimental details

**Materials.** Tetrachloroauric(III) acid ($HAuCl_4 \cdot 4H_2O$, >99.9% metals basis), 4-*tert*-butylbenzenethiol (TBBTH, 99%), diphenylphosphine oxide ($O{=}PHPh_2$), tetraoctylammonium bromide (TOAB, ≥98.0%), sodium borohydride ($NaBH_4$, 99.8%), sulfuric acid (98%), acetonitrile, dichloromethane (DCM), methanol, tetrahydrofuran (THF), and toluene were purchased from Shanghai Chemical Reagent Co., Ltd. Nafion 117 solution (5 wt%), cesium acetate (>99%), and sodium hydroxide (>99%) were purchased from Sigma-Aldrich; ethanol (>99.7%), hydrochloric acid (36%), nitric acid (>90%), and *n*-hexanes (>99.5%) were purchased from Beijing Chemical Works; Vulcan XC-72 carbon powders were obtained from Cabot. The ultrapure water (resistivity 18.2 MΩ cm) used in all experiments was produced by a Milli-Q NANO pure water system.

The $Au_{52}(TBBT)_{32}$ (abbreviated as **$Au_{52}$**) nanocluster was synthesized according to the previous work.[1]

**Stability test.** 3 mg of $Au_{52}(HOPPh_2)_8(OPPh_2)_4(TBBT)_{16}$ (abbreviated as **$Au_{52}$-PAP**) or **$Au_{52}$** was dissolved in DCM (10 mL). Upon addition of 0.5 mL of $H_2O_2$ (30 wt%), UV-Vis-NIR spectra were collected after 4−48 h.

## 2. Characterization

All UV-Vis-NIR absorption spectra were acquired in the 200–1100 nm range using a Cary3500 spectrophotometer (Agilent). Electrospray ionization (ESI) mass spectra were recorded with a Waters Q-TOF mass spectrometer using a Z-spray source. For the positive-ion mode detection, the sample was first dissolved in toluene (~0.5 mg/mL) and then diluted (3:1 v/v) with a methanol solution containing 50 mmol of cesium acetate. For the negative-ion mode detection, the sample was directly dissolved in toluene (~0.2 mg/mL). The sample was then infused into the chamber at 5 μL/min. The source temperature was maintained at 70 °C, the spray voltage was 2.20 kV, and the cone voltage was adjusted to 60 V. The single-crystal X-

ray diffraction data were collected with a Bruker D8 VENTURE CMOS PHOTON 100 diffractometer with a Helios MX multilayer monochromator using Cu Kα radiation (λ = 1.54178 Å). The infrared (IR) spectra were recorded on a Bruker Vertex 70v FT-IR spectrometer.

The XPS surface investigation was carried out on a Thermo ESCALAB 250Xi system, and the spectra were analyzed using the Thermo Scientific Avantage Data System software. Inductively coupled plasma atomic emission spectroscopy (ICP-AES), obtained on a Perkin-Elmer Optima 6300DV spectrometer, was used to measure the gold content in the electrocatalysts.

$^{1}$H NMR, $^{31}$P NMR, and $^{1}$H−$^{1}$H COSY spectra were recorded on a Bruker 400 (400 MHz) spectrometer in $CDCl_3$. Chemical shifts were quoted in parts per million (ppm) referenced to 7.26 ppm of $CHCl_3$.

**Table S1. Crystal Data and Structure Refinement for $Au_{52}$-PAP**

| | |
|---|---|
| Identification code | **$Au_{52}$-PAP** |
| CCDC number | 2177432 |
| Empirical formula | $C_{304}H_{336}Au_{52}O_{12}P_{12}S_{16}$ |
| Formula weight | 15308.57 |
| Temperature/K | 213.00 |
| Crystal system | Triclinic |
| Space group | *P*-1 |
| *a*/Å | 23.785(3) |
| *b*/Å | 31.611(5) |
| *c*/Å | 32.599(5) |
| α/° | 89.823(6) |
| β/° | 80.125(5) |
| γ/° | 76.464(5) |
| Volume/$Å^3$ | 23459(6) |
| Z | 2 |
| $\rho_{calc}$ g/$cm^3$ | 2.167 |
| $\mu$/$mm^{-1}$ | 21.288 |
| F(000) | 13600 |
| Radiation | CuKα (λ = 1.54178) |
| 2$\theta$ range for data collection/° | 7.434 to 53.000 |
| Index ranges | -28<=h<=28, -37<=k<=36, -38<=l<=36 |
| Reflections collected | 168801 |
| Independent reflections | 81387 [$R_{int}$ = 0.2024] |
| Data/restraints/parameters | 81387/4258/3095 |
| Goodness-of-fit on $F^2$ | 1.245 |
| Final *R* indexes [I >= 2$\sigma$ (I)] | $R_1$ = 0.1153, $wR_2$ = 0.2361 |
| Final R indexes [all data] | $R_1$ = 0.2730, $wR_2$ = 0.2923 |
| Largest diff. peak/hole/e$Å^{-3}$ | 5.022/-4.292 |

40% sulfuric acid could be replaced by a nitric acid or hydrochloric acid aqueous solution in the synthesis of **Au$_{52}$-PAP**.

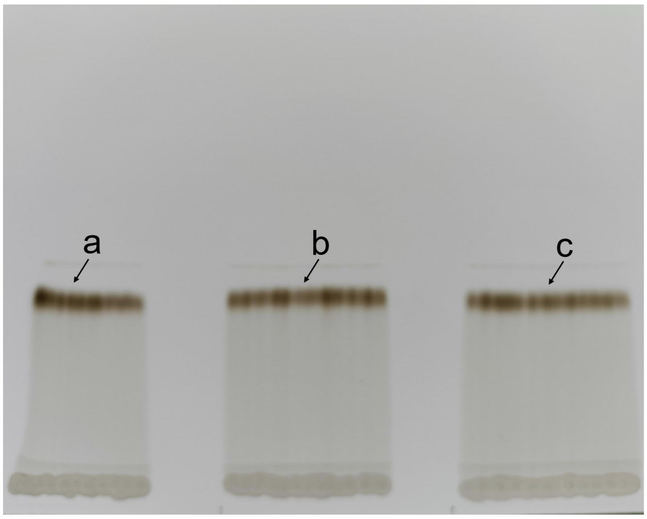


**Figure S1.** TLC plates of **Au$_{52}$-PAP** (a) and gold nanoclusters obtained with nitric acid (b) or hydrochloric acid (c).

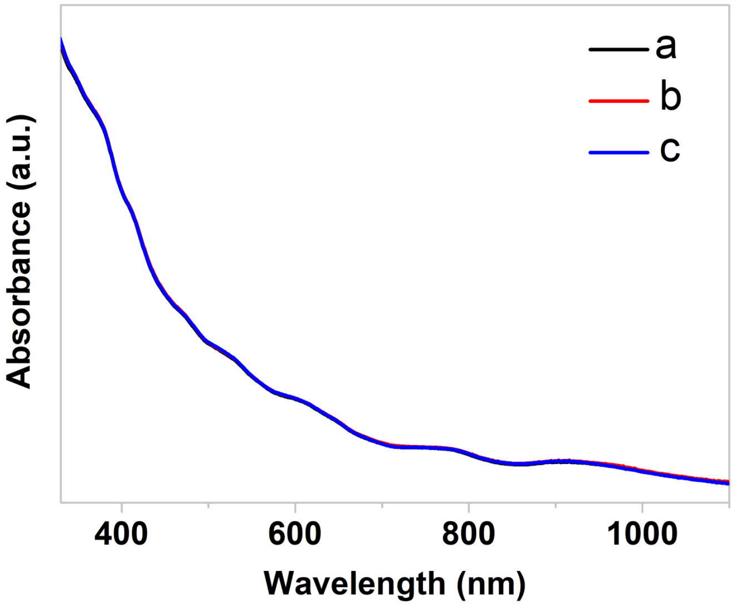


**Figure S2.** UV-Vis-NIR absorption spectra of **Au$_{52}$-PAP** (a) and gold nanoclusters obtained with nitric acid (b) or hydrochloric acid (c).

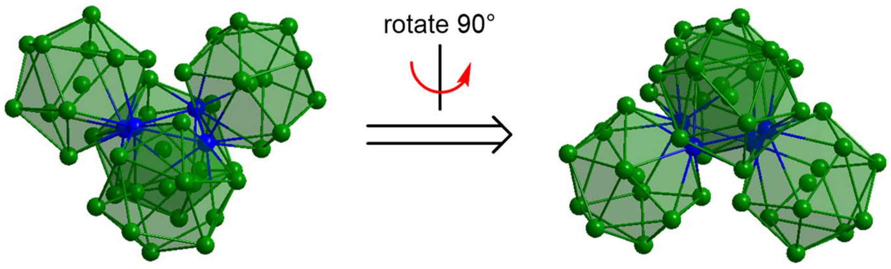


**Figure S3.** 3D “puckered” shape in the kernel of **$Au_{52}$-PAP**.

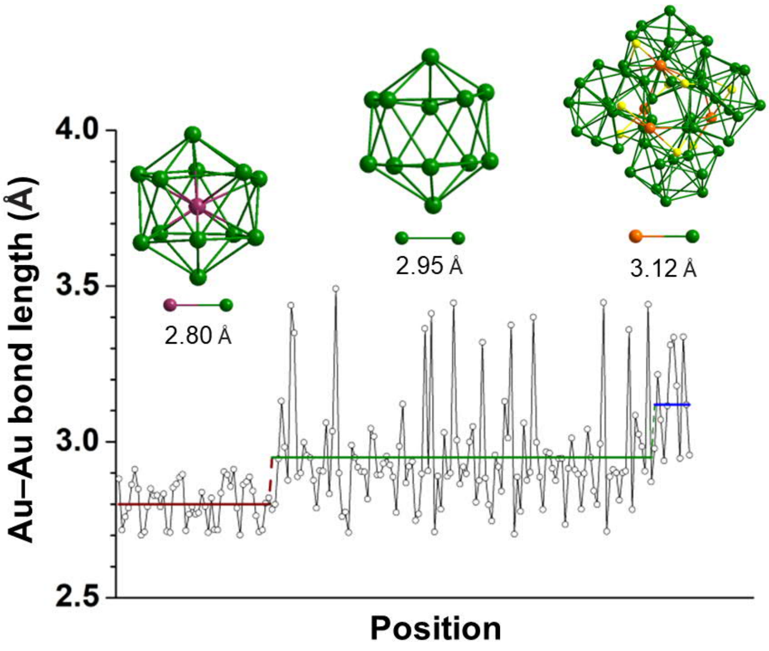


**Figure S4.** The Au–Au bonds lengths in the kernel of **$Au_{52}$-PAP**: between the central atom and the peripheral atom; between the icosahedral peripheral atoms; between the Au atom in the staples and the peripheral atoms (from left to right). Color labels: yellow, S; others, Au.

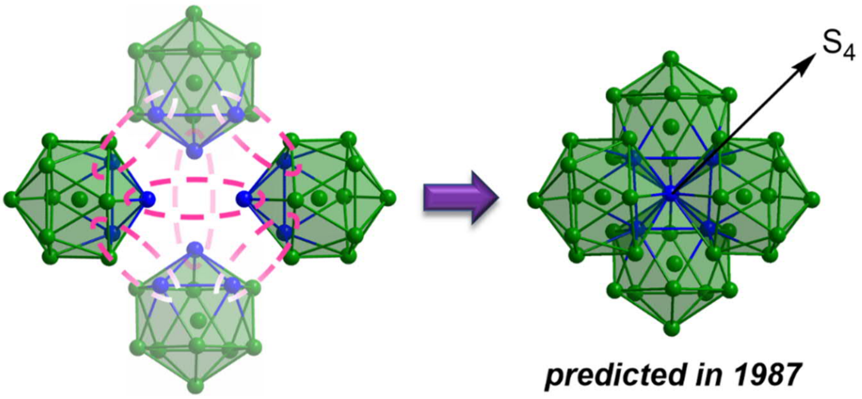


**Figure S5.** Four icosahedral $Au_{13}$ units were expected to share six vertex gold atoms to give an $Au_{46}$ kernel. The shared vertex gold atoms are highlighted in blue.

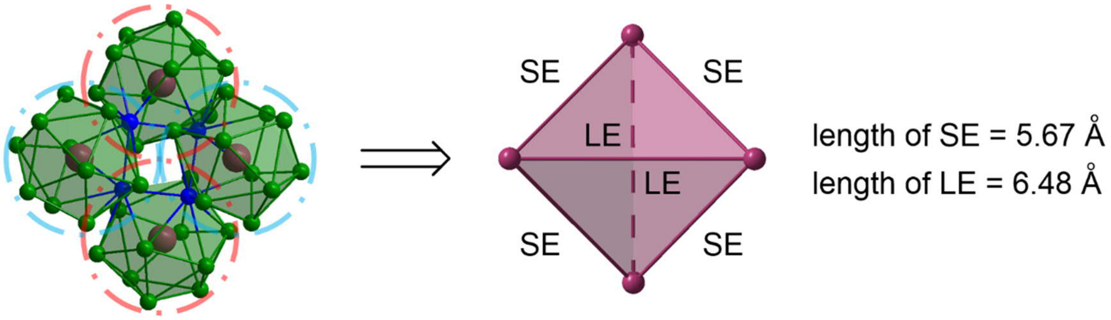


**Figure S6.** Connecting the centers of four icosahedral $Au_{13}$ units in two opposite local configurations of protecting ligands to generate a distorted tetrahedron with four short edges and two long edges. SE, short edge; LE, long edge.

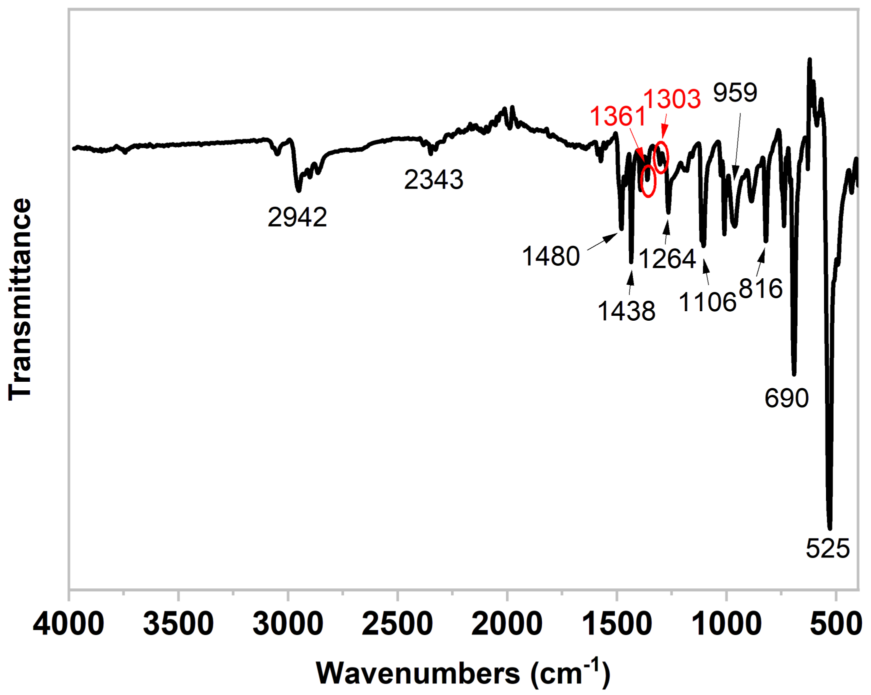


**Figure S7.** Full IR spectrum of **$Au_{52}$-PAP**.

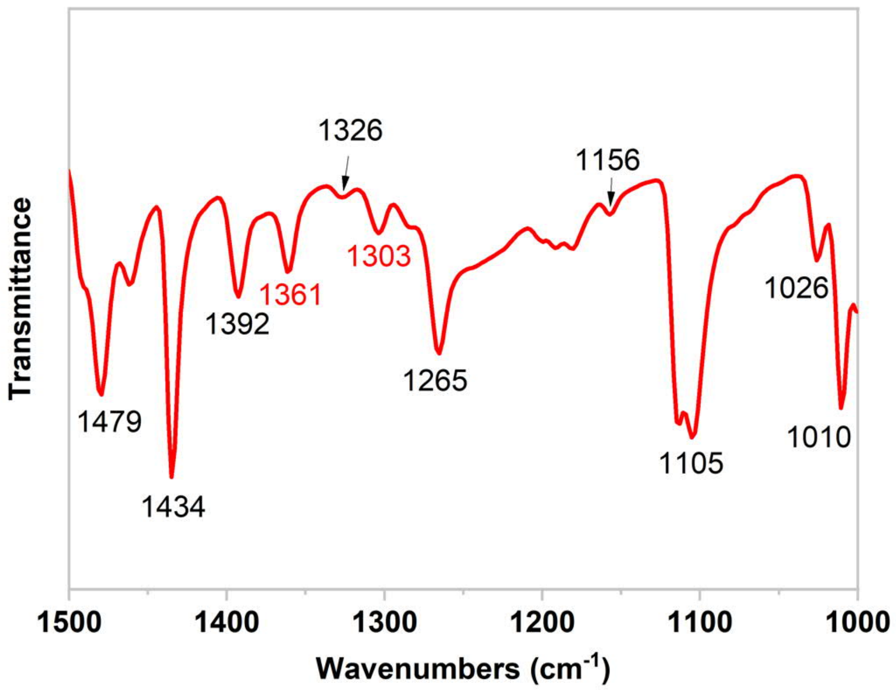


**Figure S8.** IR spectrum of **$Au_{52}$-PAP** in the wavenumber range of 1500–1000 $cm^{-1}$. The peaks at 1361 and 1303 $cm^{-1}$ could be assigned to δ-(O•••H•••O) and γ-(O•••H•••O), respectively.[8]

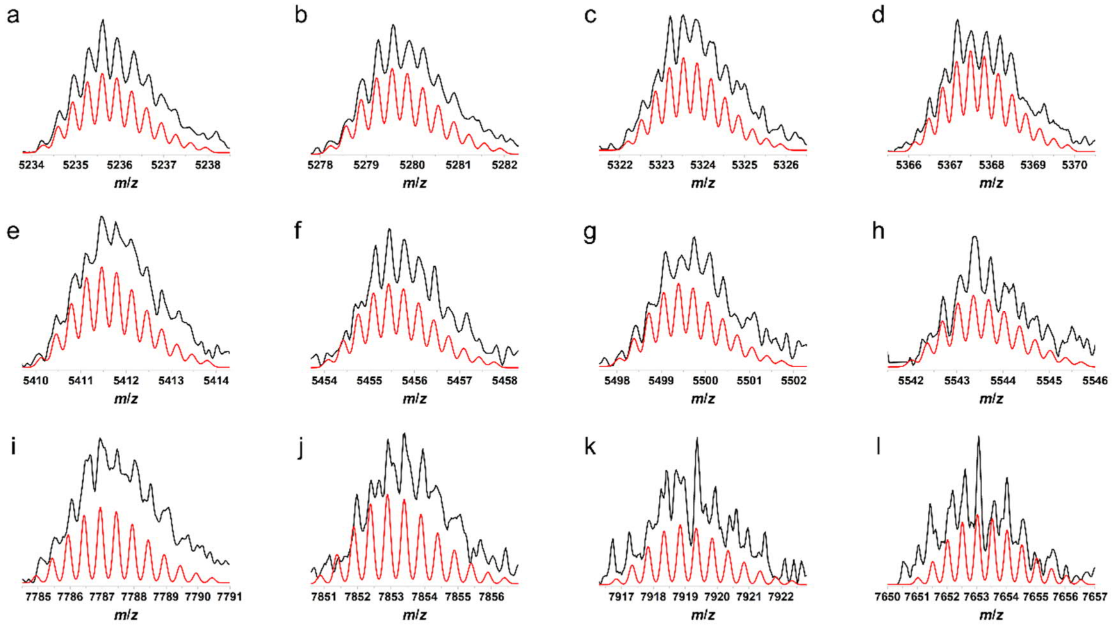


**Figure S9.** Comparison of the experimental (black lines) and simulated (red lines) isotope distribution patterns of $[M + 3Cs]^{3+}$ (a), $[M – H + 4Cs]^{3+}$ (b), $[M – 2H + 5Cs]^{3+}$ (c), $[M – 3H + 6Cs]^{3+}$ (d), $[M – 4H + 7Cs]^{3+}$ (e), $[M – 5H + 8Cs]^{3+}$ (f), $[M – 6H + 9Cs]^{3+}$ (g), $[M – 7H + 10Cs]^{3+}$ (h), $[M + 2Cs]^{2+}$ (i), $[M – H + 3Cs]^{2+}$ (j), $[M – 2H + 4Cs]^{2+}$ (k), and $[M – 2H]^{2–}$ (l). M = $Au_{52}(HOPPh_2)_8(OPPh_2)_4(TBBT)_{16}$.

**Table S2. ESI-MS of $Au_{52}$-PAP**

| Exp. (*m*/*z*) | Cal. (*m*/*z*) | Molecular formula |
|---|---|---|
| | **Positive Mode** | |
| 5236.61 | 5235.60 | $[Au_{52}(HOPPh_2)_8(OPPh_2)_4(TBBT)_{16} + 3Cs]^{3+}$ |
| 5280.58 | 5279.56 | $[Au_{52}(HOPPh_2)_8(OPPh_2)_4(TBBT)_{16} – H + 4Cs]^{3+}$ |
| 5324.53 | 5323.53 | $[Au_{52}(HOPPh_2)_8(OPPh_2)_4(TBBT)_{16} – 2H + 5Cs]^{3+}$ |
| 5368.52 | 5367.49 | $[Au_{52}(HOPPh_2)_8(OPPh_2)_4(TBBT)_{16} – 3H + 6Cs]^{3+}$ |
| 5412.47 | 5411.46 | $[Au_{52}(HOPPh_2)_8(OPPh_2)_4(TBBT)_{16} – 4H + 7Cs]^{3+}$ |
| 5456.44 | 5455.43 | $[Au_{52}(HOPPh_2)_8(OPPh_2)_4(TBBT)_{16} – 5H + 8Cs]^{3+}$ |
| 5500.74 | 5499.39 | $[Au_{52}(HOPPh_2)_8(OPPh_2)_4(TBBT)_{16} – 6H + 9Cs]^{3+}$ |
| 5544.37 | 5543.36 | $[Au_{52}(HOPPh_2)_8(OPPh_2)_4(TBBT)_{16} – 7H + 10Cs]^{3+}$ |
| 7787.94 | 7786.94 | $[Au_{52}(HOPPh_2)_8(OPPh_2)_4(TBBT)_{16} + 2Cs]^{2+}$ |
| 7854.38 | 7852.89 | $[Au_{52}(HOPPh_2)_8(OPPh_2)_4(TBBT)_{16} – H + 3Cs]^{2+}$ |
| 7920.38 | 7918.84 | $[Au_{52}(HOPPh_2)_8(OPPh_2)_4(TBBT)_{16} – 2H + 4Cs]^{2+}$ |
| | **Negative Mode** | |
| 7654.07 | 7653.03 | $[Au_{52}(HOPPh_2)_8(OPPh_2)_4(TBBT)_{16} – 2H]^{2–}$ |

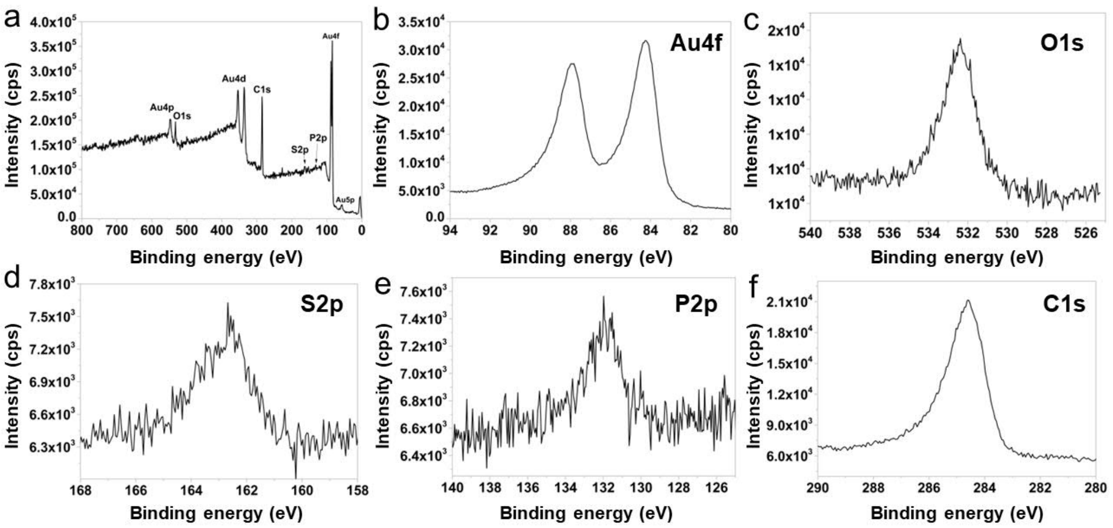


**Figure S10.** XPS spectra of **Au52-PAP**. (a) Survey spectrum of **Au52-PAP**. (b–f) High-resolution XPS spectra of Au4f, O1s, S2p, P2p, and C1s.

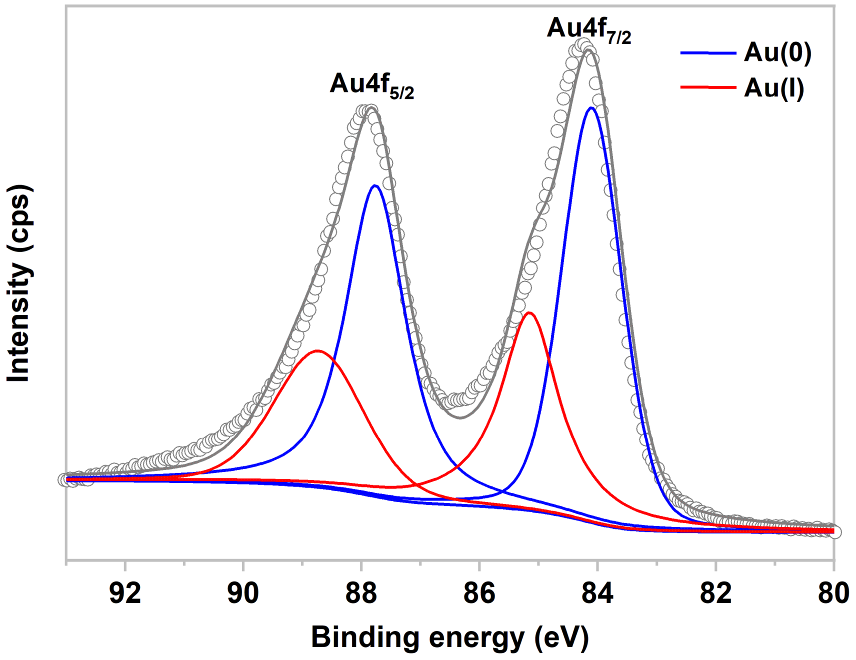


**Figure S11.** High-resolution XPS spectrum of Au4f indicating the presence of Au(0) and Au(I).

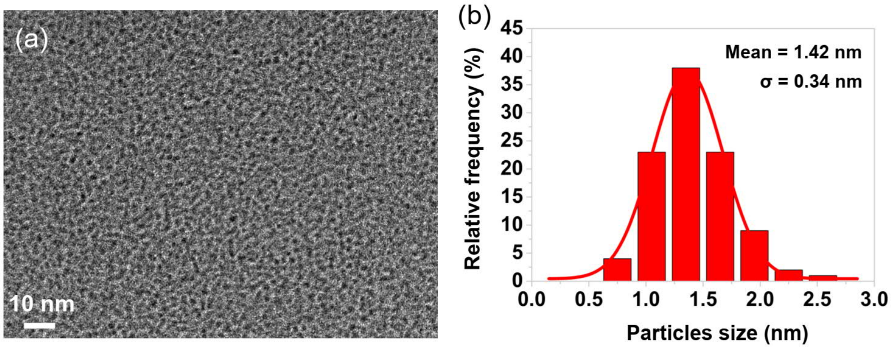


**Figure S12.** TEM images (a) and particle size histogram (b) of the **$Au_{52}$-PAP** nanoclusters.

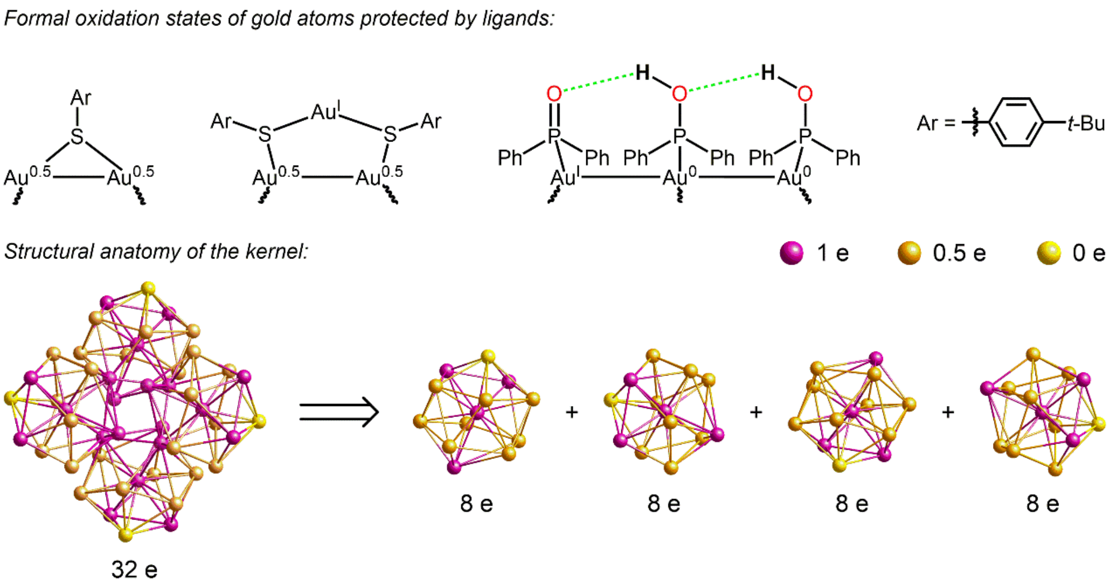


**Figure S13.** Valence electron counting in the kernel of **$Au_{52}$-PAP**. Color labels of Au having different valence electrons: 1 electron, magenta; 0.5 electron, light orange; 0 electron, yellow.

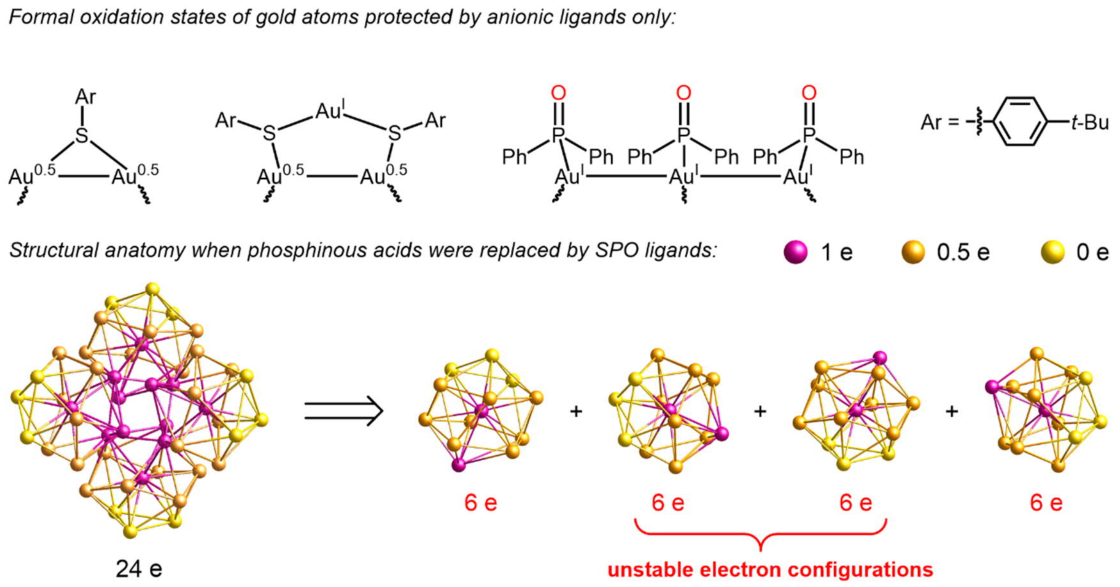


**Figure S14.** Valence electron counting in the kernel when all the phosphinous acids were replaced by phosphinito ligands. Color labels of Au having different valence electrons: 1 electron, magenta; 0.5 electron, light orange; 0 electron, yellow.

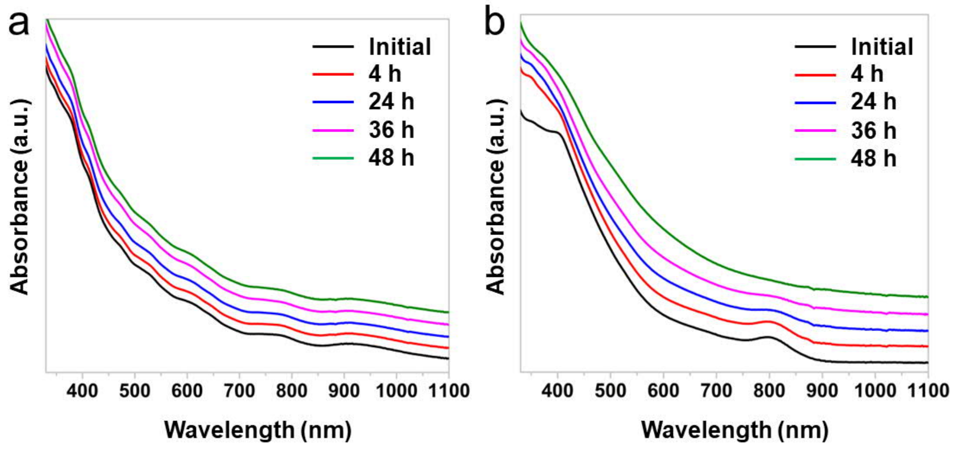


**Figure S15.** The antioxidant capacity of **$Au_{52}$-PAP** (a) and **$Au_{52}$** (b) monitored by UV-Vis-NIR spectroscopy.

## 3. Electrochemical measurements

The electrocatalysts were prepared by dispersing the **$Au_{52}$-PAP** or **$Au_{52}$** nanocluster and carbon powders in *n*-hexanes upon stirring for 6 h. Then, the homogenized electrocatalysts were collected by centrifugation and dried at room temperature in a vacuum. The accurate gold contents in the loaded samples were determined by ICP-AES. To be more specific, the determined amount of the loaded samples was first dissolved by a freshly-prepared aqua regia under vigorous stirring for 12 h. The mixture was then centrifuged at 8000 rpm for 5 min. The acquired centrifugate was diluted to a volume of 10 mL for ICP-AES testing. The gold mass loading in the electrocatalysts was eventually determined to be 5 wt% for the **$Au_{52}$-PAP** nanocluster and 4.3 wt% for the **$Au_{52}$** nanocluster, respectively.

**Table S3. Gold Nanocluster-Based Electrocatalysts for ORR**

| Catalyst | Electrolyte | Onset potential (V vs. RHE) | Half-wave potential (V vs. RHE) |
|---|---|---|---|
| $Au_{52}$-PAP | 0.1 M KOH | 0.90 | 0.69 |
| Au cluster/graphene oxide[2] | 0.1 M KOH | ~0.87 | ~0.77 |
| $Au_{11}$ nanocluster[3] | 0.1 M KOH | ~0.87 | ~0.67 |
| Au-BNNS/Au[4] | 0.1 M KOH | 0.65 | NA |
| Au sphere[5] | 0.1 M KOH | 0.71 | ~0.63 |
| Gold nanoclusters coated on carbon nanosheets[6] | 0.1 M KOH | 0.90 | ~0.8 |

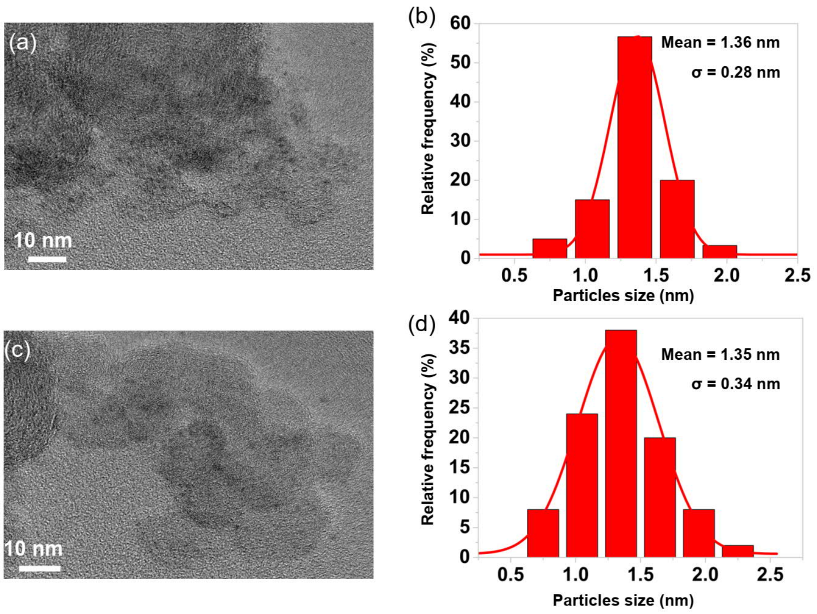


**Figure S16.** TEM images and particle size histogram of the loaded **$Au_{52}$-PAP** nanoclusters before (a, b) and after (c, d) the accelerated durability test for ORR.

## 4. Density functional theory calculations

Calculations of UV-Vis-NIR absorption spectra and Kohn−Sham molecular orbitals: Gaussian 16 package[7] was used to obtain the optimized geometry using density functional theory with the Perdew-Burke-Ernzerhof (PBE)[8] formulation within the generalized gradient approximation. The pseudopotential basis set LANL2DZ and all-electron def2-SVP were used for Au atoms and other atoms (H, C, O, S, and P), respectively. Since the gold cluster is large and the direct computation of the absorption spectra with the standard TD-DFT model is prohibitively costly, the simplified Grimme's sTDA method[9] implemented in Orca 4.2.1[10] was used to compute the spectra using the same functional and basis sets as above. Kohn−Sham orbital analysis was performed to identify the atomic orbital contribution to each MO and corresponding energies using the Multiwfn 3.8 program.[11,12]

Computational method for the Gibbs free energy of the gold cluster: we have employed the Gaussian 16 package[7] to perform all density functional theory (DFT) calculations using the Perdew-Burke-Ernzerhof (PBE)[8] formulation within the generalized gradient approximation. To save computational cost, we simplified protecting ligands from $HOPPh_2$, $OPPh_2$, and TBBT to $HOPMe_2$, $OPMe_2$, and SH, respectively. All structures were relaxed until the forces were less than 0.02 eV/Å. This R-group simplification of ligands is a commonly used approach to reduce the computational cost without affecting the interfacial bond strength. The Gibbs free energy was evaluated by the formula:

$$\Delta G = \Delta E + \Delta ZPE + \Delta \int C_p dT - T\Delta S$$

where $\Delta E$ is the adsorption energy of OOH*, O*, and OH* intermediates. $\Delta ZPE$ is the change of the zero-point energy, $C_p$ is the constant-pressure heat capacity, and $\Delta S$ is the entropy change. The projected density of states (PDOS) and the d-band centers were analyzed using Multiwfn 3.8 program.

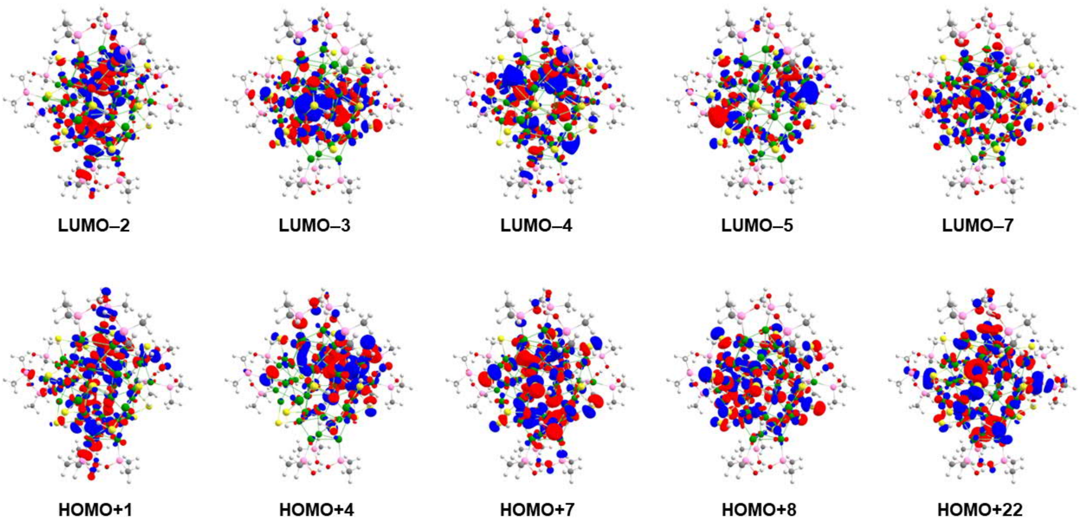


**Figure S17.** Selected frontier molecular orbitals of **$Au_{52}$-PAP**.

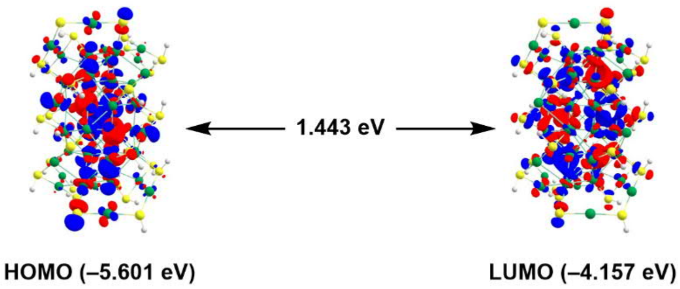


**Figure S18.** HOMO and LUMO distributions of **$Au_{52}$**.

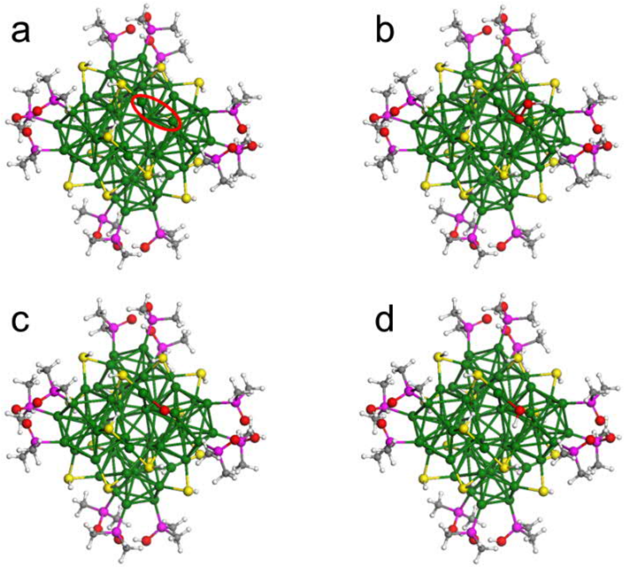


**Figure S19.** (a) Removal of the thiolate ligand (closer to the PAP ligands) from a –SH–Au–SH– staple for exposing the highly active gold center on **$Au_{52}$-PAP** to provide activated gold catalyst **$Au_{52}$-PAP-S1**. (b) Adsorption of OOH* to **$Au_{52}$-PAP-S1**. (c) Adsorption of O* to **$Au_{52}$-PAP-S1**. (d) Adsorption of OH* to **$Au_{52}$-PAP-S1**. Color labels: green, Au; yellow, S; magenta, P; red, O; grey, C; white, H.

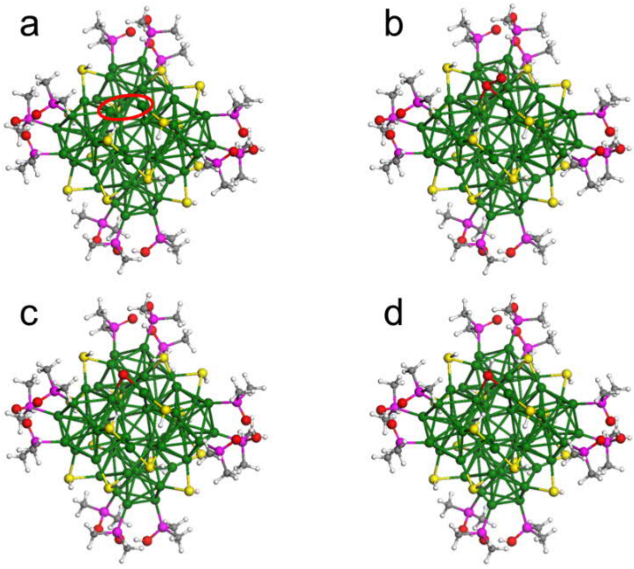


**Figure S20.** (a) Removal of the thiolate ligand (closer to other thiolate ligands) from a –SH–Au–SH– staple for exposing the highly active gold center on **$Au_{52}$-PAP** to provide activated gold catalyst **$Au_{52}$-PAP-S2**. (b) Adsorption of OOH* to **$Au_{52}$-PAP-S2**. (c) Adsorption of O* to **$Au_{52}$-PAP-S2**. (d) Adsorption of OH* to **$Au_{52}$-PAP-S2**. Color labels: green, Au; yellow, S; magenta, P; red, O; grey, C; white, H.

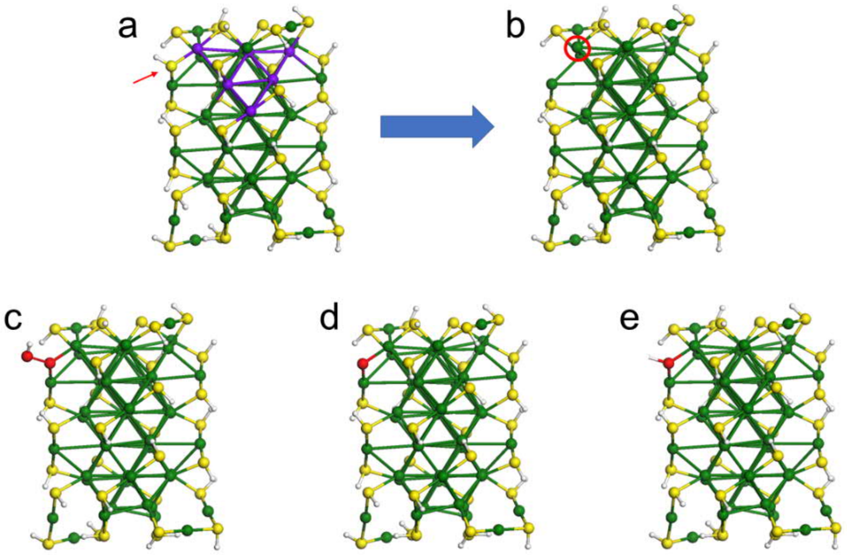


**Figure S21.** (a, b) Removal of a bridging thiolate ligand from a –Au–SH–Au– motif on the (111) surface of **$Au_{52}$** to provide activated gold catalyst **$Au_{52}$-S**. (c) Adsorption of OOH* to **$Au_{52}$-S**. (d) Adsorption of O* to **$Au_{52}$-S**. (e) Adsorption of OH* to **$Au_{52}$-S**. Color labels: yellow, S; red, O; white, H; others, Au.

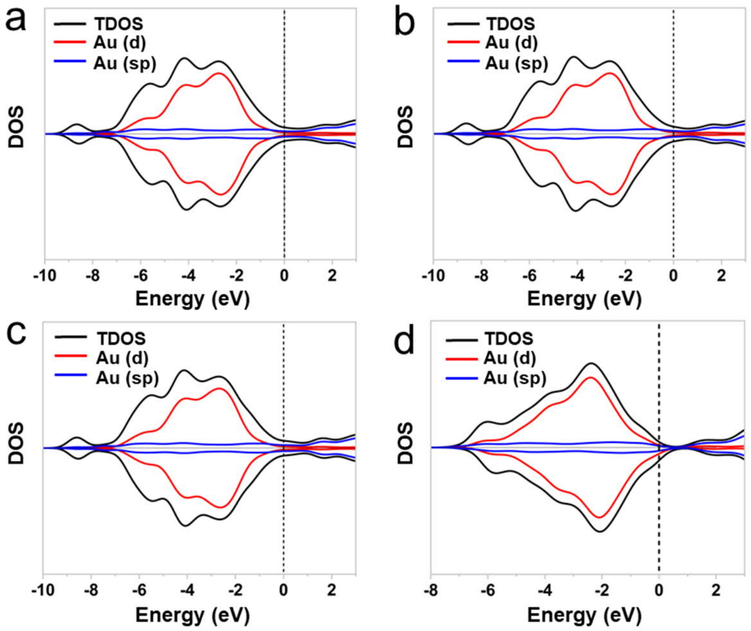


**Figure S22.** Density of states for **$Au_{52}$-PAP-P** (a), **$Au_{52}$-PAP-S1** (b), **$Au_{52}$-PAP-S2** (c), and **$Au_{52}$-S** (d).

## 5. References


(1) Zeng, C.; Chen, Y.; Liu, C.; Nobusada, K.; Rosi, N. L.; Jin, R. Gold Tetrahedra Coil Up: Kekulé-like and Double Helical Superstructures. *Sci. Adv.* **2015**, *1*, e1500425.

(2) Yin, H.; Tang, H.; Wang, D.; Gao, Y.; Tang, Z. Facile Synthesis of Surfactant-Free Au Cluster/Graphene Hybrids for High-Performance Oxygen Reduction Reaction. *ACS Nano* **2012,** *6*, 8288−8297.

(3) Chen, W.; Chen, S. Oxygen Electroreduction Catalyzed by Gold Nanoclusters: Strong Core Size Effects. *Angew. Chem., Int. Ed.* **2009,** *48*, 4386−4389.

(4) Elumalai, G.; Noguchi, H.; Lyalin, A.; Taketsugu, T.; Uosaki, K. Gold Nanoparticle Decoration of Insulating Boron Nitride Nanosheet on Inert Gold Electrode toward an Efficient Electrocatalyst for the Reduction of Oxygen to Water. *Electrochem. Commun.* **2016,** *66*, 53−57.

(5) Balasubramanian, S.; Sheelam, A.; Ramanujam, K.; Dhamodharan, R. Green, Seed-Mediated Synthesis of Au Nanowires and Their Efficient Electrocatalytic Activity in Oxygen Reduction Reaction. *ACS Appl. Mater. Interfaces* **2017,** *9*, 28876−28886.

(6) Wang, Q.; Wang, L.; Tang, Z.; Wang, F.; Yan, W.; Yang, H.; Zhou, W.; Li, L.; Kang, X.; Chen, S. Oxygen Reduction Catalyzed by Gold Nanoclusters Supported on Carbon Nanosheets. *Nanoscale* **2016,** *8*, 6629−6635.

(7) Frisch, M. J.; Trucks, G. W.; Schlegel, H. B.; Scuseria, G. E.; Robb, M. A.; Cheeseman, J. R.; Scalmani, G.; Barone, V.; Petersson, G. A.; Nakatsuji, H.; Li, X.; Caricato, M.; Marenich, A. V.; Bloino, J.; Janesko, B. G.; Gomperts, R.; Mennucci, B.; Hratchian, H. P.; Ortiz, J. V.; Izmaylov, A. F.; Sonnenberg, J. L.; Williams; Ding, F.; Lipparini, F.; Egidi, F.; Goings, J.; Peng, B.; Petrone, A.; Henderson, T.; Ranasinghe, D.; Zakrzewski, V. G.; Gao, J.; Rega, N.; Zheng, G.; Liang, W.; Hada, M.; Ehara, M.; Toyota, K.; Fukuda, R.; Hasegawa, J.; Ishida, M.; Nakajima, T.; Honda, Y.; Kitao, O.; Nakai, H.; Vreven, T.; Throssell, K.; Montgomery Jr., J. A.; Peralta, J. E.; Ogliaro, F.; Bearpark, M. J.; Heyd, J. J.; Brothers, E. N.; Kudin, K. N.; Staroverov, V. N.; Keith, T. A.; Kobayashi, R.; Normand, J.; Raghavachari, K.; Rendell, A. P.; Burant, J. C.; Iyengar, S. S.; Tomasi, J.; Cossi, M.; Millam, J. M.; Klene, M.; Adamo, C.; Cammi, R.; Ochterski, J. W.; Martin, R. L.; Morokuma, K.; Farkas, O.; Foresman, J. B.; Fox, D. J. *Gaussian 16 Rev. A.03*, Wallingford, CT, 2016.

(8) Perdew, J. P.; Burke, K.; Ernzerhof, M. Generalized Gradient Approximation Made Simple. *Phys. Rev. Lett.* **1996**, *77*, 3865−3868.

(9) Grimme, S. A Simplified Tamm-Dancoff Density Functional Approach for the Electronic Excitation Spectra of very Large Molecules. *J. Chem. Phys.* **2013**, *138*, 244104.

(10) Neese, F. Software Update: the ORCA Program System, Version 4.0. *WIREs Comput. Mol. Sci.* **2018**, *8*, e1327.

(11) Lu, T.; Chen, F. Calculation of Molecular Orbital Composition. *Acta Chim. Sinica* **2011**, *69*, 2393−2406.

(12) Lu, T.; Chen, F. Multiwfn: A Multifunctional Wavefunction Analyzer. *J. Comput. Chem.* **2012**, *33*, 580−592.
